# Clinical efficacy of different blood purification modes on severe acute pancreatitis: a systematic review and network meta-analysis

**DOI:** 10.3389/fmed.2026.1767153

**Published:** 2026-05-29

**Authors:** Peihong Li, Hao Tian, Po Huang, Xiaolei Fang

**Affiliations:** Department of Emergency, Dongfang Hospital, Beijing University of Chinese Medicine, Beijing, China

**Keywords:** blood purification, CRRT, hemofiltration, hemoperfusion, network meta-analysis, severe acute pancreatitis

## Abstract

**Objective:**

To evaluate the clinical efficacy of different blood purification modes on Severe Acute Pancreatitis (SAP) based on a network meta-analysis, providing new insights for clinical treatment of SAP.

**Methods:**

Computerized searches were conducted in English databases (PubMed, Embase, Cochrane Library, Web of Science) and Chinese databases (CNKI, VIP, Wangfang Data, CBM) for clinical trials published through December 1, 2025, evaluating various blood purification modes for SAP. Study quality was assessed using the RoB 2.0 (Cochrane risk of bias tool for randomized trials, version 2). Meta-analysis was performed using STATA 18.0 software under the frequentist framework with a random-effects model. Surface Under the Cumulative Ranking Curve (SUCRA) values were used to rank the efficacy of interventions. Publication bias was assessed using funnel plots, and loop inconsistency was examined for closed loops in the evidence network.

**Results:**

A total of 45 randomized controlled trials involving 3,707 patients were included. The network meta-analysis showed that regarding the primary outcome of Acute Physiology and Chronic Health Evaluation (APACHE II) scores, hemoperfusion + continuous renal replacement therapy ranked as the most effective intervention. For secondary outcomes:continuous renal replacement therapy plus plasma exchange and hemoperfusion combined with continuous renal replacement therapy were among the most effective interventions for reducing amylase (AMS), C-reactive protein (CRP), interleukin-6 (IL-6). Hemoperfusion combined with continuous renal replacement therapy demonstrated the greatest improvement in triglyceride (TG) levels in patients with hyperlipidemic SAP, and in renal function (Scr). However, in terms of mortality outcomes, blood purification did not show a trend toward benefit compared with conventional therapy. And for certain outcomes, loop inconsistency was detected and heterogeneity could not be effectively resolved, warranting cautious interpretation.

**Conclusion:**

Hemoperfusion combined with continuous renal replacement therapy demonstrateds more satisfactory therapeutic effects and significantly reduces amylase levels, improves inflammatory response, renal function, and disease severity in SAP patients, and effectively lowers TG levels in those with hyperlipidemic SAP, but hemoperfusion combined with continuous renal replacement therapy does not significantly decrease mortality. These findings provide evidence-based guidance for clinical decision-making, though further high-quality trials are required to confirm the optimal blood purification strategy.

**Systematic review registration:**

https://www.crd.york.ac.uk/prospero/, identifier CRD420261403366.

## 1 Introduction

Acute pancreatitis (AP) refers to an acute abdomen caused by abnormal activation of pancreatic enzymes that produces digestion of the pancreas itself and surrounding organs and is mainly characterized by local inflammatory reactions in pancreas, which can even lead to organ dysfunction (1). Severe acute pancreatitis (SAP) is defined by the presence of persistent (more than 48 hours) organ failure, accounting for 5–25% of all AP cases (2–4). Mortality rate of SAP can reach 20–30% due to its serious condition and rapid progression (5). Hyperlipidemic Severe Acute Pancreatitis refers to SAP accompanied by triglyceride (TG) levels greater than 11.1 mmol/L (6).

When treating SAP, clinical guidelines suggest that intensive care unit (ICU) should be admitted as early as possible once multiple organ failure is found (1, 2). Treatment of SAP mainly lies in surgical treatment and medical comprehensive treatment, while surgical treatment is primarily indicated for patients with intestinal obstruction, pancreatic and peripancreatic necrosis causing infection (2). With the in-depth studies on SAP, individualized comprehensive treatment is gradually conducted in some cases. Medical management includes correcting electrolyte imbalances, nutritional support, inhibiting pancreatic secretion, preventing and treating infections, abdominal lavage, blood purification, endoscopic therapy, and traditional Chinese medicine treatment (7), among which blood purification extracts the blood from the body to purify various impurities through various ways, so as to achieve the effect of controlling sepsis, excessive inflammatory state and other life-threatening diseases (8). Although the good effect of blood purification on SAP has been proved by several clinical trials (9), there still lacks evidence for the horizontal comparison of the therapeutic effect of various blood purification modes, which is not conducive to the clinical selection of specific methods for SAP. Therefore, by using network meta-analysis, this study compares the clinical efficacy of SAP combined with different blood purification modes on the basis of conventional treatment, in order to provide a more comprehensive evidence-based reference for the clinical selection of effective blood purification methods.

## 2 Methods

This review was performed in accordance with the Preferred Reporting Items for Systematic Reviews and Meta-Analyses (PRISMA) guidelines (10, 11), with PROSPERO registration (CRD420251163895, registered on October 8, 2025).

### 2.1 Search strategy

A comprehensive electronic database search strategy was constructed to identify randomized controlled trials (RCTs) reporting the clinical efficacy of different blood purification modes on SAP. The systematic search was performed in PubMed, Embase, the Cochrane library, Web of Science, CNKI, VIP, CBM, Wangfang Data using a combination of relevant medical subject heading (MeSH) terms and text words including severe acute pancreatitis, hemoperfusion, hemodialysis, hemodiafiltration, plasma exchange, continuous renal replacement therapy (CRRT), with the Boolean search terms “OR” and “AND” (Supplementary Table 1). Trials published between the database establishment and December 1, 2025 were considered eligible.

Following the systematic search, two authors (LPH and TH) independently screened all papers for eligibility. Studies were initially screened by title and abstract, and subsequently by full text if they met the predetermined inclusion criteria. Any inconsistency and disagreements were discussed by the researchers and a consensus was reached with the opinion of a fourth researcher (FXL), if necessary. Following study recruitment, the respective data of all included studies were extracted via Microsoft Excel. A third reviewer (HP) independently assessed and verified all data extraction.

### 2.2 Inclusion criteria

Incorporate published studies on different blood purification methods for patients with severe acute pancreatitis (SAP), and the diagnosis of SAP must comply with the 2012 revision of the Atlanta classification and definitions by international consensus (3). These studies should involve interventions such as blood purification, continuous renal replacement therapy, hemodialysis, hemoperfusion, and plasma exchange. amylase (AMS), Interleukin-6 (IL-6), C-reactive protein (CRP), Serum creatinine (Scr), Acute Physiology and Chronic Health Evaluation (APACHE II) scores, Triglyceride (TG) and mortality should be used as outcome indicators. The language should be set to both Chinese and English. Additionally, they must be randomized controlled trials (RCTs).

### 2.3 Exclusion criteria

Conference papers, animal experiments, case reports, dissertations, those with insufficient design rigor, missing data, or flawed or erroneous statistical applications, duplicate publications of the same set of data, and analyses of experimental data from public databases were excluded.

### 2.4 Data extraction

Two researchers independently extracted data. Disagreements were resolved through discussion or consultation with a third party to reach consensus. We recorded each trial’s authors, publication date, number of participants in the intervention and control groups, gender distribution, and intervention measures. Outcome measures included AMS, CRP, IL-6, Scr, APACHE II scores, TG, and mortality. The change in APACHE II score before and after treatment was selected as the primary outcome measure in this study. First, the APACHE II score is a comprehensive quantitative indicator of overall disease severity, encompassing acute physiology score (12 physiological parameters), age points, and chronic health status (12). It reflects multi-organ function and systemic physiological homeostasis; thus, the dynamic change in score objectively captures the evolution of the patient’s clinical status and is more representative than any single indicator. Second, as previously described, acute pancreatitis is a dynamically evolving disease process, during which worsening physiological assessment scores, identify patients at high risk for complications or fatal outcomes (13). Hence, the change in APACHE II score not only quantifies therapeutic response but also carries prognostic value. Finally, mortality was not chosen as the primary outcome because many previous studies lacked complete documentation of deaths, and the observation periods varied considerably across studies—pooling mortality data directly could introduce bias and compromise comparability and reliability. In the setting of extremely elevated serum TG levels, TG-rich chylomicrons are hydrolyzed by pancreatic lipase, releasing large amounts of free fatty acids (FFAs), which can induce local acidosis and an inflammatory cascade, thereby further aggravating pancreatic necrosis. Therefore, TG reduction serves as a disease-specific indicator of therapeutic response in hyperlipidemic SAP (HTG-SAP). For non-hyperlipidemic SAP, TG was not assessed, because hypertriglyceridemia is not a primary pathophysiological feature of the disease. For continuous variables, we recorded the mean and standard deviation (SD) at baseline and at the end of the intervention period. To minimize the impact of baseline differences on treatment effect estimates, we calculated and used the mean change from baseline for each group in the subsequent network meta-analysis. The specific conversion formula can be found in the Supplementary Figure 1. For dichotomous outcomes, such as mortality and treatment failure rate, which cannot be converted into change values, we only performed inconsistency testing and heterogeneity analysis in the subsequent network meta-analysis.

### 2.5 Risk of bias assessment

The RoB 2.0 (Cochrane risk of bias tool for randomized trials, version 2) was used to assess the risk of bias of the included RCTs (14). Two reviewers independently assessed the risk of bias in the studies we finally select. The following domains of bias were detected: Randomization process, deviations from intended interventions, missing outcome data, measurement of the outcome, and selection of the reported result. If there were discrepancies, the discrepancies were resolved through discussion with a third team member until we reached a consensus.

### 2.6 Statistical analysis

Statistical analysis was performed in this study under the frequentist framework using STATA 18.0. All outcome measures were combined using a random-effects model: Risk ratio (RR) was used as effect size for dichotomous variables, continuous variables were presented as mean difference (MD). If the units are inconsistent but there is a clear conversion relationship, the units should be unified first, and MD should still be used as the effect size for pooling. If different measurement scales or units were used, resulting in data that cannot be combined through direct unit conversion, the standardized mean difference (SMD) should be selected as the effect size to eliminate the impact of heterogeneity. And then 95% confidence interval (CI) was calculated. In network meta-analysis, network package was used to construct the network evidence diagram for the comparison of interventions, the area under the cumulative ranking probability curve was calculated to rank the efficacy of each intervention probability, in order to determine the relative optimal scheme. It is worth mentioning that all the outcome indicators included in this study are negative indicators, meaning that a higher measured value indicates a worse clinical treatment effect. Therefore, in the SUCRA ranking, we adopt the “smaller is better” ranking principle: the intervention with the smallest effect size is defined as the optimal solution. A higher SUCRA value indicates that the intervention ranks higher and has a better efficacy in controlling adverse outcomes. A funnel plot is drawn to assess publication bias. If a closed loop of evidence was formed in the network, we assessed loop inconsistency to evaluate the coherence between direct and indirect estimates.

## 3 Results

### 3.1 Search results and study selection

**FIGURE 1:**
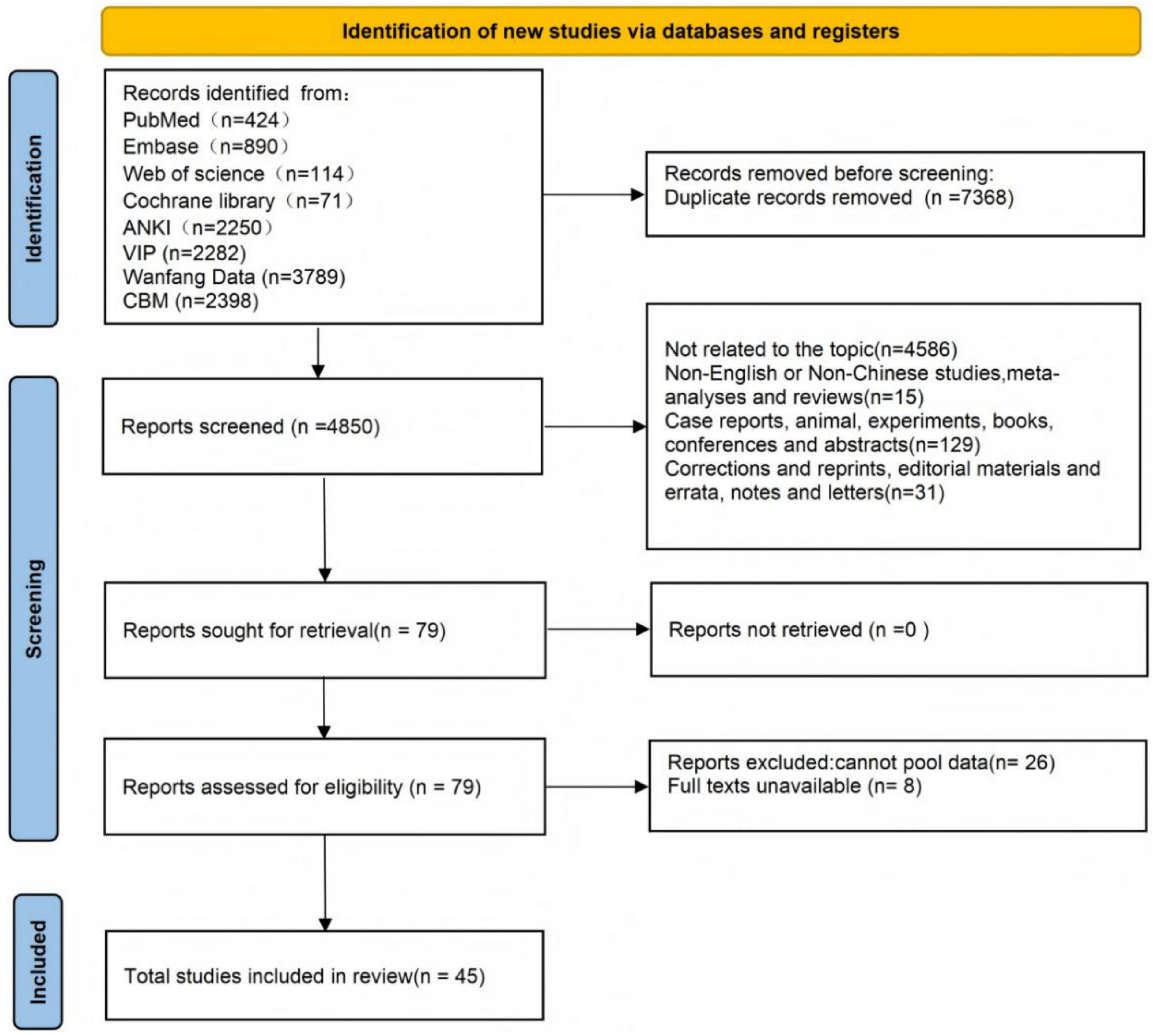
Research selection flowchart.

As of December 1, 2025, a total of 12,218 articles were identified in the initial literature search. After eliminating duplicates (*n* = 7,368) and adhering to specific inclusion and exclusion criteria, Forty-five studies (involving 3,707 participants and conducted between 2014 and 2024) were finally included in the network Meta-analysis, including 41 of Chinese articles (15–55), and 4 of English articles (56–59). The literature search and study selection process is showed in Figure 1. We meant to compare the following different blood purification modes: continuous renal replacement therapy (C), continuous renal replacement therapy + plasma exchange (C + PE), hemodialysis (HD), hemodiafiltration (HDF), hemofiltration (HF), hemoperfusion + continuous renal replacement therapy (HP + C), hemoperfusion + hemodialysis (HP + HD), hemoperfusion + hemodiafiltration (HP + HDF), non-blood purification group (N), plasma exchange (PE), plasma exchange + hemodialysis (PE + HD). The basic information included in the study is provided in Supplementary Table 2.

**FIGURE 2:**
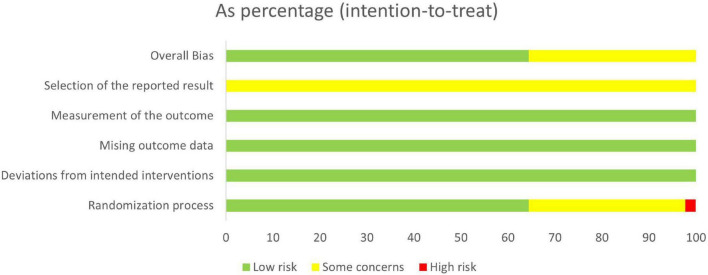
Risk of bias graph.

Among the 45 included RCTs, for bias arising from the randomization process, 25 explicitly stated the use of a random number table for randomization, 1 explicitly stated the use of the double-ball lottery method for randomization, 2 explicitly stated the use of computer-generated randomization, all of these were rated as “low risk.” 1 explicitly proposes the use of odd and even hospitalization numbers for randomization, which falls under the category of quasi-randomization methods and is rated as “high risk.” The remaining 15 studies only indicated random assignment without specifying the randomization method, thus rated as “some concerns.” For Bias due to deviations from intended interventions, all studies were conducted according to the pre-specified intervention allocation, rated as “low risk.” For bias due to missing outcome data, no articles reported loss to follow-up, thus all were rated “low risk.” For bias in measurement of the outcome data, the measurement methods for all outcome indicators in the literature are appropriate, hence they are rated as “low risk.” For bias in selection of the reported result, all the literature comes from publicly available search databases, and it is impossible to determine whether there is a selective reporting bias in the results. Therefore, it is rated as “some concerns.” For overall bias, 16 studies are rated as “some concerns,” and other 29 studies are rated as “low-risk.” The risk of bias assessment for included studies is shown in Figure 2.

### 3.2 Inconsistency test results

The inconsistency model was used for overall inconsistency testing, and the node splitting method was employed for local inconsistency testing. The *P*-values for the overall inconsistency testing of each outcome indicator were all greater than 0.05, indicating no significant inconsistency (specific *P*-values can be found in Supplementary Table 3). The node splitting method revealed that the direct comparison evidence and indirect comparison evidence for each outcome indicator were consistent (*P* > 0.05), with specific *P*-values available in Supplementary Figure 2. All outcomes formed closed loops, therefore we conducted an evaluation of loop inconsistency to evaluate the coherence between direct and indirect estimates. When loop inconsistency was assessed for any closed loop in the network, A *P*-value > 0.05 for the inconsistency factor (IF) suggested no statistically significant disagreement between direct and indirect evidence. An IF value less than 1 indicated low heterogeneity within the loop, whereas an IF greater than 1 suggested a potential trend toward inconsistency. If significant inconsistency or heterogeneity was detected, we performed conventional pairwise meta-analyses using STATA 18.0 to explore its sources and conducted a leave-one-out analysis. If there were studies at high risk of bias in the included literature, we conducted sensitivity analyses by excluding them and performed a reanalysis to compare the results before and after removal.

### 3.3 Network meta-analysis of pancreatic function

#### 3.3.1 AMS (U/L)

**FIGURE 3:**
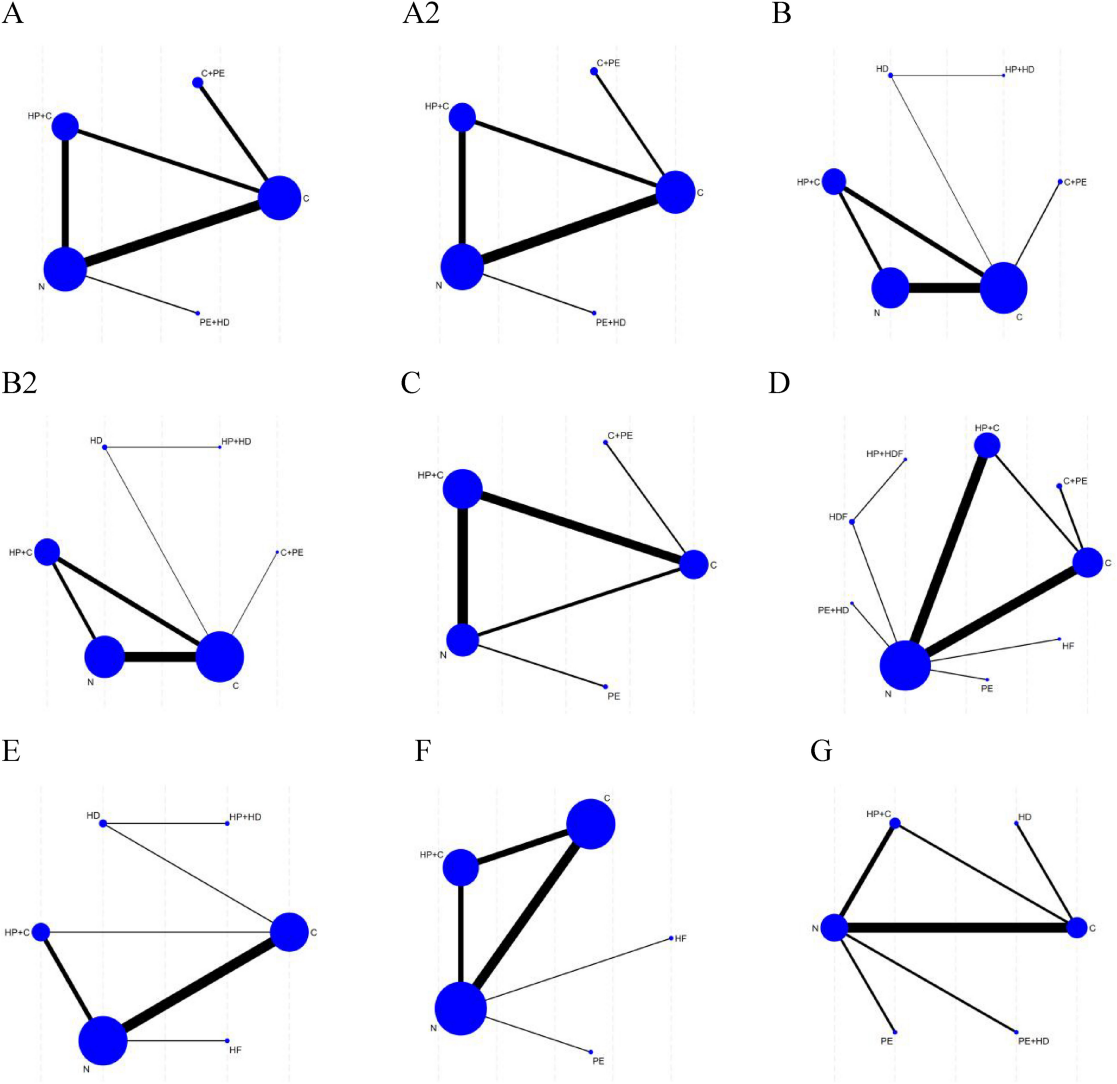
Network plots of clinical efficacy of different blood purification modes on SAP. **(A)** AMS, **(A2)** AMS excluding quasi-randomization method, **(B)** CRP, **(B2)** CRP excluding quasi-randomization method, **(C)** IL-6, **(D)** Scr, **(E)** Apache II, **(F)** TG, **(G)** Mortality. Continuous renal replacement therapy (C), continuous renal replacement therapy + plasma exchange (C + PE), hemodialysis (HD), hemodiafiltration (HDF), hemofiltration (HF), hemoperfusion + continuous renal replacement therapy (HP + C), hemoperfusion + hemodialysis (HP + HD), hemoperfusion + hemodiafiltration (HP + HDF), Non-blood purification group (N), plasma exchange (PE), plasma exchange + hemodialysis (PE + HD).

**FIGURE 4:**
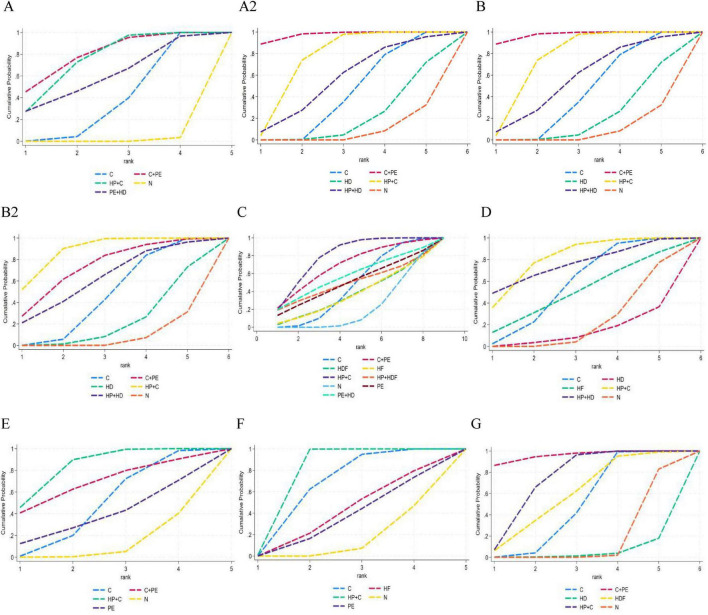
SUCRA cumulative probability curve illustrating the clinical efficacy of different blood purification modes on SAP. **(A)** AMS, **(A2)** AMS excluding quasi-randomization method, **(B)** CRP, **(B2)** CRP excluding quasi-randomization method, **(C)** IL-6, **(D)** Scr, **(E)** Apache II, **(F)** TG, **(G)** Mortality. Continuous renal replacement therapy (C), continuous renal replacement therapy + plasma exchange (C + PE), hemodialysis (HD), hemodiafiltration (HDF), hemofiltration (HF), hemoperfusion + continuous renal replacement therapy (HP + C), hemoperfusion + hemodialysis (HP + HD), hemoperfusion + hemodiafiltration (HP + HDF), Non-blood purification group (N), plasma exchange (PE), plasma exchange + hemodialysis (PE + HD).

Nineteen studies recorded the AMS values of 1,557 patients before and after different interventions, involving four blood purification methods. Network plots of clinical efficacy of different blood purification modes in improving AMS Scores in SAP Patients is shown in Figure 3A. Global inconsistency testing and the node-splitting method revealed no significant inconsistency (*P* > 0.05 for all; see Supplementary Table 3 and Supplementary Figure 2. For brevity, the same conclusion applies to all other outcome indicators, and no further statements on inconsistency will be repeated in the subsequent sections), supporting the use of a network meta-analysis (NMA). The NMA results showed that C + PE (MD = 2.16, 95% CI: 0.96, 3.36), HP + C (MD = 2.31, 95% CI: 1.11, 3.50), showing statistically significant differences (*P* < 0.05). Meanwhile, CRRT showed significant advantages compared to PE + HD, with a statistically significant difference (*P* < 0.05). No statistically significant differences were observed in pairwise comparisons between other blood purification methods (*P* > 0.05). Some of the intervention measures in this study formed a closed loop, and we conducted a loop inconsistency test on the closed loop. The results showed that the non-blood purification group combined with HP + CRRT and CRRT formed a closed loop [IF = 74.841, 95% CI (0.00, 385.96), *P* = 0.637], indicating that there was no statistically significant difference between direct and indirect evidence. However, the absolute value of IF was large, indicating a possible inconsistency trend, which may be due to heterogeneity within the intervention measures. Therefore, we performed conventional pairwise meta-analyses to explore heterogeneity. For the comparison of Hemoperfusion combined with CRRT versus CRRT alone, significant heterogeneity was observed (*I*^2^ = 95.0%; Supplementary Figure 3). A leave-one-out sensitivity analysis revealed that the study by Yilong et al. (46) was the primary source of heterogeneity [WMD = −14.31, 95% CI (−24.06, −4.56)], After its exclusion, heterogeneity decreased substantially (I^2^ decreased from 95.0 to 0.0%) as detailed in Supplementary Figure 4. Notably, this study had the shortest intervention duration (2 days) compared with all other included studies (≥ 3 days), which may explain its divergent effect size and its substantial contribution to the overall heterogeneity. For the comparison between the Hemoperfusion combined with CRRT and Non-blood purification group, significant heterogeneity was also present (*I*^2^ = 98.4%; Supplementary Figure 5). However, leave-one-out sensitivity analysis failed to substantially reduce heterogeneity. The study by Sun et al. (57) had the greatest influence [WMD = −881.45, 95% CI (−990.10, −772.80)], with I^2^ remaining at 91.4% after exclusion, see Supplementary Figure 6, possibly due to its small sample size. Similarly, for the comparison between the CRRT and Non-blood purification group, significant heterogeneity was found (*I*^2^ = 96.9%; Supplementary Figure 7), and leave-one-out analysis could not effectively reduce it. The study by Bo et al. (16) had the largest impact [WMD = −53.78, 95% CI (45.67, 61.89)], with I^2^ decreasing to 93.6% after exclusion (Supplementary Figure 8). This may be partly attributable to the fact that the study did not clearly report the duration of the intervention, the lack of clarity regarding intervention duration makes it difficult to assess whether an adequate treatment course was delivered, which may have influenced the effect size and contributed to the overall heterogeneity. Among the 29 included studies, one used a quasi-randomization method (33). After excluding this study, we re-analyzed the data and found no substantial changes in heterogeneity (Supplementary Figure 9), network plots (Figure 3A2), SUCRA values (Supplementary Table 5), SUCRA cumulative probability curves (Figure 4A2), or treatment rankings (Supplementary Table 14), indicating that its impact on the overall results was negligible. According to the SUCRA rankings, C + PE (79.4%) and HP + C (74.3%) showed significantly higher efficacy in reducing AMS. It should be noted that due to failed loop inconsistency tests and the inability to reduce heterogeneity through leave-one-out analysis, the current SUCRA values are relatively unstable; therefore, the ranking results should be interpreted with caution. Detailed SUCRA values (MD, 95% CI) are presented in Supplementary Table 4, the SUCRA cumulative probability curves are shown in Figure 4A, and the probability rankings are provided in Supplementary Table 14.

### 3.4 Network meta-analysis of inflammatory parameters

#### 3.4.1 CRP

Thirty studies recorded the CRP values of 2,354 patients before and after different interventions, involving five blood purification methods. Due to substantial heterogeneity in measurement units across studies, the standardized mean difference (SMD) with 95% confidence intervals (CIs) was used as the effect measure for subsequent analyses. Network plots of clinical efficacy of different blood purification modes in improving CRP Scores in SAP Patients is shown in Figure 3B. The NMA results showed that HP + C (SMD = 2.88, 95% CI 2.19–3.57), C + PE (SMD = 4.30, 95% CI 2.68–5.91), and CRRT alone (SMD = 1.39, 95% CI 0.87–1.90) were associated with statistically significant improvements in CRP reduction (*P* < 0.05). Furthermore, both HP + C and C + PE demonstrated significant advantages over CRRT alone (*P* < 0.05). Loop-specific inconsistency tests were performed for closed loops in the network. The results showed no significant inconsistency between direct and indirect evidence [IF = 0.074, 95% CI (0.00, 1.34), *P* = 0.910], and the IF value < 1 indicated low heterogeneity within the loop. Among the 29 included studies, one used a quasi-randomization method (33). After excluding this study, we re-analyzed the data and found no substantial changes in inconsistency [IF = 0.074, 95% CI (0.00, 1.34), *P* = 0.910], network plots (Figure 3B2) SUCRA values (Supplementary Table 7), SUCRA cumulative probability curves (Figure 4B2), or treatment rankings (Supplementary Table 14), indicating that its impact on the overall results was negligible. According to the SUCRA rankings, C + PE (97.4%) and HP + C (75.1%) showed significantly higher efficacy in reducing CRP. Detailed SUCRA values (SMD, 95% CI) are presented in Supplementary Table 6, the SUCRA cumulative probability curves are shown in Figure 4B, and the probability rankings are provided in Supplementary Table 14.

#### 3.4.2 IL-6 (ng/L)

Twenty-seven studies recorded the IL-6 values of 2120 patients before and after different interventions, involving eight blood purification methods. Network plots of clinical efficacy of different blood purification modes in improving AMS Scores in SAP Patients is shown in Figure 3C. The NMA results showed that HP + C (MD = 2.14, 95% CI: 1.34, 2.95), C + PE (MD = 3.33, 95% CI: 1.28, 5.38), showing statistically significant differences (*P* < 0.05). No statistically significant differences were observed in pairwise comparisons between other blood purification methods (*P* > 0.05). Loop-specific inconsistency tests were performed for closed loops in the network. The results showed that the non-blood purification group combined with HP + C and C formed a closed loop [IF = 25.859, 95% CI (0.00, 276.21), *P* = 0.544]. We performed conventional pairwise meta-analyses to explore heterogeneity. For the comparison of HP + C versus CRRT alone, significant heterogeneity was observed (*I*^2^ = 94.6%; Supplementary Figure 10). However, as only two studies were included in this comparison, it was not possible to conduct a leave-one-out sensitivity analysis. For the comparison between the HP + C and Non-blood purification group, significant heterogeneity was also present (*I*^2^ = 99.6%; Supplementary Figure 11). However, leave-one-out sensitivity analysis failed to substantially reduce heterogeneity. The study by Libing et al. (28) had the greatest influence [WMD = −139.20, 95% CI (−153.24, −125.16)], with I^2^ remaining at 98.9% after exclusion, see Supplementary Figure 12, The study by Huang Libing was identified as a major contributor to the observed heterogeneity. This may be attributable to its exclusion criteria: unlike most other included studies, this trial explicitly excluded patients with infectious diseases. Given that infectious complications are common in SAP and are associated with higher baseline inflammatory levels and more pronounced responses to blood purification, the exclusion of such patients may have resulted in a study population with systematically different characteristics. This selection bias likely influenced the effect size and contributed disproportionately to the overall heterogeneity. Similarly, for the comparison between the CRRT and Non-blood purification group, significant heterogeneity was found (*I*^2^ = 97.2%; Supplementary Figure 13), and leave-one-out analysis could not effectively reduce it. The study by Nan (32) had the largest impact [WMD = −4.99, 95% CI (−6.04, −3.94)], with I^2^ decreasing to 88.6% after exclusion (Supplementary Figure 14). This may be attributable to differences in inclusion criteria: while most other studies only required imaging evidence of pancreatitis for enrollment, Li Nan additionally required imaging evidence of severity. This stricter criterion likely resulted in the inclusion of patients with more severe disease compared to other trials. According to the SUCRA rankings, C + PE (86.5%) was associated with the greatest reduction in IL-6, followed by HP + C (67.9%). However, it is worth noting that the loop inconsistency test was not passed, and heterogeneity could not be effectively reduced by leave-one-out sensitivity analysis. Consequently, the stability of these SUCRA estimates is limited, and the ranking results warrant cautious interpretation. Detailed SUCRA values (MD, 95% CI) are presented in Supplementary Table 8, the SUCRA cumulative probability curves are shown in Figure 4C, and the probability rankings are provided in Supplementary Table 14.

### 3.5 Network meta-analysis of renal function

#### 3.5.1 Scr (μmol/L)

Seventeen studies recorded the Scr values of 1,302 patients before and after different interventions, involving five blood purification methods. Network plots of clinical efficacy of different blood purification modes in improving Scr Scores in SAP Patients is shown in Figure 3D. The NMA results showed that HP + C (MD = 136.18, 95% CI: 6.27, 266.09) showing statistically significant differences (*P* < 0.05). In addition, HP + HD showed a significant advantage over HD alone, and the difference was statistically significant (*P* < 0.05). No statistically significant differences were observed in pairwise comparisons between other blood purification methods (*P* > 0.05). Loop-specific inconsistency tests were performed for closed loops in the network. The results showed that the Non-blood purification group combined with HP + C and C formed a closed loop [IF = 65.284, 95% CI (0.00, 276.21), *P* = 0.544]. We performed conventional pairwise meta-analyses to explore heterogeneity. For the comparison between the CRRT and Non-blood purification group, significant heterogeneity was found (*I*^2^ = 99.6%; Supplementary Figure 15), and leave-one-out analysis could not effectively reduce it. The study by Guo et al. (59) had the largest impact [WMD = −210.33, 95% CI (−217.37, −203.29)] with I^2^ decreasing to 70.2% after exclusion (Supplementary Figure 16). This may be attributable to its exclusion criteria: unlike most other included studies, H. Guo explicitly excluded patients with renal failure. Since renal function at baseline is a critical determinant of the potential for Scr improvement following blood purification, the exclusion of patients with renal failure likely resulted in a study population with lower baseline Scr levels and, consequently, a reduced capacity for further reduction. This selection bias may have systematically influenced the effect size and contributed substantially to the overall heterogeneity. No significant heterogeneity was detected between the HP + C and Non-blood purification group, with *I*^2^ = 0.0% (see Supplementary Figure 17). In the comparison of HP + C versus CRRT alone, heterogeneity could not be evaluated due to the inclusion of only one study (see Supplementary Figure 18). According to the SUCRA rankings, HP + C (61.2%) was associated with the greatest reduction in Scr. However, it is worth noting that the loop inconsistency test was not passed, and heterogeneity could not be effectively reduced by leave-one-out sensitivity analysis. Consequently, the stability of these SUCRA estimates is limited, and the ranking results warrant cautious interpretation. Detailed SUCRA values (MD, 95% CI) are presented in Supplementary Table 9, the SUCRA cumulative probability curves are shown in Figure 4D, and the probability rankings are provided in Supplementary Table 14.

### 3.6 Network meta-analysis of critical illness score

#### 3.6.1 APACHE II scores

Twenty-two studies recorded the APACHEII Scores values of 1,543 patients before and after different interventions, involving seven blood purification methods. Network plots of clinical efficacy of different blood purification modes in improving APACHEII Scores in SAP Patients is shown in Figure 3E. The NMA results showed that HP + C (MD = 1.83, 95% CI: 1.38, 2.29]), and CRRT alone (MD = 0.94, 95% CI: 0.56, 1.32) were associated with statistically significant improvements in APACHEII Scores reduction (*P* < 0.05). In addition, HP + C showed a significant advantage over PE, HF, and C, with all differences reaching statistical significance (*P* < 0.05). Loop-specific inconsistency tests were performed for closed loops in the network. The results showed no significant inconsistency between direct and indirect evidence [IF = 0.546, 95% CI (0.00, 4.10), *P* = 0.763], and the IF value < 1 indicated low heterogeneity within the loop. According to the SUCRA rankings, HP + C (99.0%) was associated with the greatest reduction in APACHEII Scores, followed by CRRT alone (64.4%). Detailed SUCRA values (MD, 95% CI) are presented in Supplementary Table 10, the SUCRA cumulative probability curves are shown in Figure 4E, and the probability rankings are provided in Supplementary Table 14.

### 3.7 Network meta-analysis of blood lipid levels

#### 3.7.1 TG (mmol/L)

Given that the some included studies focused on patients with Hyperlipidemic Severe Acute pancreatitis, and TG levels are a core clinical feature and therapeutic target for these patients, this study conducted an analysis using TG as an independent outcome indicator to evaluate the improvement effect of different blood purification methods on lipid metabolism disorders in Hyperlipidemic Severe Acute pancreatitis patients.

Fifteen studies recorded the TG values of 1,018 patients before and after different interventions for the treatment of Hyperlipidemic Severe Acute pancreatitis, involving four blood purification methods. Network plots of clinical efficacy of different blood purification modes in improving TG in SAP Patients is shown in Figure 3F. The NMA results showed that HP + C (MD = 2.48, 95% CI: 1.22, 3.74) was associated with statistically significant improvements in TG Scores reduction (P < 0.05). Loop-specific inconsistency tests were performed for closed loops in the network. The results showed no significant inconsistency between direct and indirect evidence [IF = 0.501, 95% CI (0.00, 11.69), *P* = 0.930], and the IF value < 1 indicated low heterogeneity within the loop. According to the SUCRA rankings, HP + C (83.7%) was associated with the greatest reduction in TG. Detailed SUCRA values (MD, 95% CI) are presented in Supplementary Table 11, the SUCRA cumulative probability curves are shown in Figure 4F, and the probability rankings are provided in Supplementary Table 14.

### 3.8 Network meta-analysis of prognosis

#### 3.8.1 Mortality

Ten studies recorded the number of deaths among 800 patients during the intervention period for various intervention measures, involving five blood purification methods. Treatment effects for dichotomous variables were expressed as risk ratios (RRs) with 95% confidence intervals. Network plots of clinical efficacy of different blood purification modes in reducing Mortality in SAP Patients is shown in Figure 3G. The current results suggest that blood purification does not reduce mortality in SAP patients compared with conventional therapy. This may be attributable to insufficient observation time and the fact that many included studies did not report mortality outcomes. Additionally, pairwise comparisons showed that HD significantly reduced mortality compared with PE + HD (HD: RR = 0.03, 95% CI: 0.00, 0.85) and HP + C (HD: RR = 0.03, 95% CI: 0.00, 0.28). Loop-specific inconsistency tests were performed for closed loops in the network. The results showed no significant inconsistency between direct and indirect evidence [IF = 1.196, 95% CI (0.00, 3.99), *P* = 0.401]. We performed conventional pairwise meta-analyses to explore heterogeneity. No significant heterogeneity was detected between the HP + C and Non-blood purification group, with *I*^2^ = 0.0% (see Supplementary Figure 19). In the comparison of HP + C versus CRRT alone, heterogeneity could not be evaluated due to the inclusion of only one study (see Supplementary Figure 20). For the comparison between the CRRT and Non-blood purification group, no significant heterogeneity was found(*I*^2^ = 0.00%; Supplementary Figure 21). It is worth noting that despite low statistical heterogeneity in direct comparisons, the network exhibited notable inconsistency (IF > 1), undermining the reliability of the network estimates. Consequently, the credibility of the SUCRA values is compromised, and the rankings should be regarded as preliminary. Detailed SUCRA values (RR, 95% CI) are presented in Supplementary Table 12, the SUCRA cumulative probability curves are shown in Figure 4G, and the probability rankings are provided in Supplementary Table 14.

### 3.9 Publication bias

**FIGURE 5:**
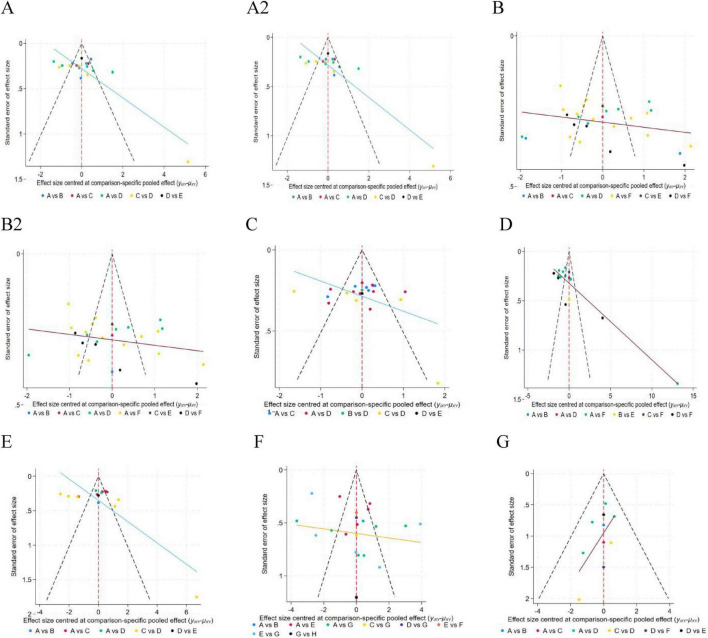
Funnel plot of studies contributing to the network for the primary outcome. **(A)** AMS, **(A2)** AMS excluding quasi-randomization method, **(B)** CRP, **(B2)** CRP excluding quasi-randomization method, **(C)** IL-6, **(D)** Scr, **(E)** Apache II, **(F)** TG, **(G)** Mortality. Continuous renal replacement therapy (C), continuous renal replacement therapy + plasma exchange (C + PE), hemodialysis (HD), hemodiafiltration (HDF), hemofiltration (HF), hemoperfusion + continuous renal replacement therapy (HP + C), hemoperfusion + hemodialysis (HP + HD), hemoperfusion + hemodiafiltration (HP + HDF), Non-blood purification group (N), plasma exchange (PE), plasma exchange + hemodialysis (PE + HD).

The publication bias assessment of the included literature was conducted using a funnel plot drawn by STATA 18.0. The results showed that the scatter plot distribution of the funnel plot was not completely symmetrical, and some studies were located outside the boundaries of the funnel plot, suggesting a possible publication bias. The funnel plot is shown in Figure 5.

## 4 Discussion

### 4.1 Summary of key findings

This network meta-analysis evaluates the therapeutic effects of different blood purification methods on SAP from six dimensions: pancreatic function, inflammatory markers, renal function, critical illness scores, lipid profile, and mortality. The study found that C + PE and HP + C showed superior efficacy in reducing AMS, CRP and IL-6. HP + C was most effective in improving Scr, APACHE II score, and TG levels, while blood purification did not show statistically significant advantages in reducing mortality. The failure of blood purification to reduce mortality in SAP patients may be due to insufficient observation time and the fact that many included studies did not report mortality outcomes. This information can assist clinicians in making choices when treating SAP.

### 4.2 Comparison with previous research

Recently, multiple blood purification therapies have been confirmed to improve various indicators of SAP, as well as to prevent the occurrence and development of systemic inflammatory response syndrome (SIRS) (3, 60). However, due to many different blood purification methods and combinations, relevant evidence on accurately select blood purification methods in clinical practice is still limited. Thus, through network meta-analysis, we conducted horizontal study to compare different blood purification modes and its combination style, in order to provide more evidence-based support for more clinical selection.

### 4.3 Mechanism of action of blood purification therapy

The pathogenesis of SAP has always been a hot spot. People used to focus on the theory of pancreatic enzyme autodigestion (61), but with the gradual deepening of research, the theory of inflammatory mediators centered on the theory of leukocyte Hyperactivation and the theory of bacterial infection play an important role in the pathogenesis of SAP (62), have also become the focus of research (61). Inflammatory factors such as IL-6 and CRP contribute to the progression of SAP, and their elevated levels are closely related to organ dysfunction (63). The decrease of IL-6 is strongly associated with better prognosis of SAP (61). Endotoxin produced by bacteria can further damage the body and induce the development of experimental severe acute pancreatitis, and metabolites can dilate blood vessels and lead to ischemic injury of the pancreas The core mechanism of HTG-induced SAP involves the hydrolysis of circulating triglyceride-rich chylomicrons by pancreatic lipase in the pancreatic microvasculature due to extreme hypertriglyceridemia. This releases abundant free fatty acids (FFAs) whose lipotoxicity directly damages acinar and endothelial cells, inducing local acidosis and an inflammatory cascade that exacerbates pancreatic necrosis (64).

Blood purification can effectively remove inflammatory mediators. Among the various modalities, CRRT removes target molecules from the blood through convection and diffusion. It offers distinct advantages in eliminating inflammatory mediators, correcting water and electrolyte imbalances, and stabilizing hemodynamics. CRRT can selectively remove inflammatory factors *in vivo* and effectively resolve the inflammatory cytokine storm (65, 66). This is attributable to the high-flux membrane used in CRRT, which limits the size of molecules that can pass through, but consequently reduces its clearance efficiency for large-molecular-weight pathogenic substances.

Hemoperfusion directs blood through a cartridge containing adsorbents, where toxins and inflammatory mediators in the blood are immobilized onto the adsorbent surface via direct adsorption. The advantages of this adsorption-based approach are fourfold. First, HP efficiently adsorbs medium and large molecular weight inflammatory mediators, including various cytokines such as IL-6 and tumor necrosis TNF-α, as well as lipopolysaccharide (LPS) and AMS (67). Secondly, in patients with SAP, disruption of the intestinal barrier can lead to secondary gut-derived endotoxemia (68). Endotoxin continuously activates the NF-kB pathway, promoting the production of IL-6 and other cytokines (69). By eliminating endotoxin, HP attenuates this positive feedback loop at its source, thereby indirectly inhibiting the persistent synthesis of CRP and IL-6. Thirdly, improvement in the APACHE II score reflects global amelioration of multiorgan function and systemic physiological homeostasis. Specifically, the marked improvement in APACHE II score observed with HP + C can be attributed to the complementary mechanisms described above: HP primarily removes medium and large molecular-weight inflammatory mediators and endotoxin, whereas CRRT predominantly clears small- and medium-sized molecules. Together, they establish a full-spectrum clearance system spanning from small to large molecules, synergistically controlling the inflammatory cascade and breaking the vicious cycle of “pancreatic injury → cytokine storm → multiorgan dysfunction.” Consequently, this comprehensive approach improves multiorgan function, which is precisely what the APACHE II score aims to capture. Fourth, the adsorbents used in HP possess hydrophobic properties, conferring a strong adsorption capacity for lipid-soluble substances such as triglyceride-rich chylomicrons (70). HP can also adsorb free triglyceride molecules and apolipoproteins to varying degrees, thereby blocking the generation and release of free fatty acids (FFAs) and subsequently reducing pancreatic injury. HP + C may contribute to the improvement of Scr through multiple mechanisms. First, by eliminating inflammatory cytokines such as IL-6, it interrupts TLR4 pathway-mediated inflammatory injury and apoptosis in renal tubular epithelial cells (HK-2) (71). Second, through the clearance of FFAs, HP + C prevents FFA-induced damage to renal microvascular endothelial cells and tubular cells, inhibits mitochondrial complex function, and mitigates renal inflammation and organ failure (72). Collectively, these mechanisms support a favorable role of HP + C in improving Scr levels.

Plasma exchange involves withdrawing whole blood, separating it into plasma and cellular components extracorporeally, discarding the plasma, and then mixing the cellular fraction with a replacement solution (e.g., fresh frozen plasma, albumin solution, and balanced electrolyte solution) before returning the mixture to the patient at the same infusion rate. This procedure achieves the goals of reducing pathological damage and eliminating pathogenic substances, rapidly and effectively removing impurities from the blood, attenuating the autodigestive capacity of trypsin on the pancreas, improving microcirculation in pancreatic tissue, and alleviating hypercoagulability. A key advantage of PE lies in its ability to non-selectively and extensively clear large molecular-weight pathogenic substances from plasma, including IL-1β, IL-6, TNF-α, and AMS. As noted previously, this is precisely what CRRT alone cannot achieve.

The synergistic effect of combining PE with CRRT in reducing levels of AMS, CRP, and IL-6 is further attributable to the continuous operation of CRRT, which maintains a stable and sustained clearance of inflammatory mediators, thereby preventing a rebound surge in inflammatory markers during the interval between two PE sessions. This rationale aligns with the early “peak concentration hypothesis” proposed by Uchino and colleagues (73), which posits that the goal of extracorporeal blood purification techniques is not to maximize the absolute quantity of mediators removed, but rather to attenuate the peak concentration of inflammatory mediators, thereby preventing them from exceeding the pathogenic threshold and consequently preserving organ function. Nevertheless, the clinical application of plasma exchange is substantially limited by its high cost and the often constrained availability of plasma resources in healthcare settings.

### 4.4 Adverse events and complications

In the included studies, several explicitly recorded the adverse reactions and complications associated with each intervention. Zhan et al. (52) found that the HP + C group had two cases of hemodialysis catheter infection, suggesting an increased risk of nosocomial infection. Jing and Juan (26) reported that the conventional treatment group had 3 cases of acute respiratory distress syndrome (ARDS), 1 case of acute encephalopathy, 4 cases of hypovolemic shock, and 3 cases of acute cardiac dysfunction; the HP + C group had 1 case of ARDS, 0 cases of acute encephalopathy, 1 case of hypovolemic shock, and 3 cases of acute cardiac dysfunction, indicating that HP + C may reduce the incidence of ARDS, acute encephalopathy, and hypovolemic shock. Lin et al. (29) observed that the conventional treatment group had 7 cases of systemic inflammatory response syndrome (SIRS), 5 cases of multiple organ dysfunction syndrome (MODS), 4 cases of multiple organ failure (MOF), and 5 cases of acute kidney injury; the HP + C group had 2 cases of SIRS, 2 cases of MODS, 2 cases of MOF, and 3 cases of acute kidney injury, also suggesting that HP + C may lower the incidence of complications. Peipei et al. (33) compared CRRT with C + PE and found that the CRRT group had 24 cases of ARDS, 21 cases of MODS, and 27 cases of pancreatic encephalopathy; the C + PE group had 4 cases of ARDS, 5 cases of MODS, and 6 cases of pancreatic encephalopathy, demonstrating that C + PE significantly reduced the occurrence of ARDS, MODS, and pancreatic encephalopathy. Guo et al. (59) reported that the conventional treatment group had 15 cases of mechanical ventilation, 11 cases of MODS, and 6 cases of ARDS; the CRRT group had 12 cases of mechanical ventilation, 6 cases of MODS, and 2 cases of ARDS, indicating that CRRT can reduce the incidence of these complications

### 4.5 Limitations of the study and future improvement measures

This study has certain limitations. Firstly, the included literature comprises some articles and RCTs that do not explicitly mention blinding and randomization methods, which may affect the overall quality and credibility of the evidence. Secondly, a potential limitation of this study is the predominance of Chinese-language articles (41 out of 45 included studies). This geographical and language concentration may limit the generalizability of our findings to other populations or healthcare systems with different genetic backgrounds, dietary habits, disease etiologies, and clinical practice patterns for SAP. Thirdly, from the evidence network diagram (Supplementary Figure 4), it can be observed that the evidence network exhibits sparsity. This may lead to direct comparison results based on a limited number of studies being more susceptible to the influence of individual study characteristics (such as small sample size, baseline risk differences), as well as the potential for insufficient power in consistency tests, thereby resulting in bias. Therefore, caution should be exercised when interpreting comparison results generated by thin lines. In addition, there were inevitable differences in baseline characteristics across the included studies, including patient demographics (e.g., age), disease severity, disease duration, intervention course, and concomitant conventional treatments. To minimize the potential impact of these baseline imbalances on the treatment effect estimates, we calculated the mean change from baseline for continuous outcomes whenever possible. Furthermore, global inconsistency tests and node-splitting analyses revealed no significant inconsistency for most outcomes, supporting the validity of the network meta-analysis. However, for certain outcomes, the loop inconsistency test was not passed, indicating potential disagreement between direct and indirect evidence within the network. Although we attempted to explore the sources of inconsistency using leave-one-out sensitivity analysis, heterogeneity could not be effectively reduced, suggesting that the observed inconsistency may stem from underlying clinical or methodological diversity among the included studies rather than being driven by a single study. Consequently, the results for these outcomes should be interpreted with caution, as the presence of inconsistency may compromise the reliability of the network meta-analysis estimates. In the future, more rigorously designed, standardized, large-sample, multinational, and multicenter randomized controlled trials should be conducted, including populations of different ethnicities and medical backgrounds, to further clarify the universal efficacy of the intervention measures and provide a stronger evidence base for clinical practice worldwide.

## 5 Conclusion

Through a network meta-analysis, we systematically evaluated different blood purification modalities for SAP. HP + C showed the most satisfactory therapeutic effect among all modalities as it significantly improved pancreatic function (AMS), inflammation, renal function, and disease severity in SAP patients, and effectively lowered TG levels in those with hyperlipidemic SAP. However, HP + C did not demonstrate a statistically significant benefit in reducing mortality. More high-quality randomized controlled trials and more rigorous evidence-based analyses are needed in the future to clarify the choice of blood purification methods. What requires particular attention is that significant heterogeneity was observed in the inconsistency tests for the loop of evidence involving AMS, IL-6, Scr and Mortality. In light of this, the research conclusions regarding these outcome measures should be treated with caution, and their reliability urgently requires further confirmation through additional clinical and evidence-based studies in the future.

## Data Availability

The original contributions presented in this study are included in the article/Supplementary material, further inquiries can be directed to the corresponding author.
