# Supplementary material for: Clinical efficacy of different blood purification modes on severe acute pancreatitis: a systematic review and network meta-analysis
Source: Front Med (Lausanne). 2026 May 29;13:1767153. doi: 10.3389/fmed.2026.1767153 (PMC13260056; doi:10.3389/fmed.2026.1767153)
Supplement: Supplementary file 1 [file Table_1.docx]

**Supplementary Table 1.** Search strategy to find potential relevant articles for inclusion in the Network meta-analysis of clinical efficacy of different blood purification modes on SAP (November 1, 2025).

| PubMed | (((((("Pancreatitis"[Mesh]) OR (Pancreatitis, AcuteORAcute PancreatitisORAcute PancreatitidesORPancreatitides, AcuteORPancreatitis, Acute EdematousORAcute Edematous PancreatitidesOREdematous Pancreatitides, AcuteOREdematous Pancreatitis, AcuteORPancreatitides, Acute EdematousORAcute Edematous PancreatitisORPeripancreatic Fat NecrosisORFat Necrosis, PeripancreaticORNecrosis, Peripancreatic FatORPeripancreatic Fat NecrosesORPancreatic Parenchymal EdemaOREdema, Pancreatic ParenchymalORPancreatic Parenchymal EdemasORParenchymal Edema, PancreaticORPancreatic Parenchyma with Edema[Title/Abstract])) OR (("Hemofiltration"[Mesh]) OR (HemofiltrationsORArteriovenous HemofiltrationORArteriovenous HemofiltrationsORHemofiltration,Arteriovenous OR Venovenous Hemofiltration ORHemofiltration,Venovenous OR Venovenous Hemofiltrations[Title/Abstract]))) OR (("Hemoperfusion"[Mesh]) OR (Hemoperfusions OR Hemosorption ORHemosorptions[Title/Abstract]))) OR (("Continuous Renal Replacement Therapy"[Mesh]) OR (CRRT TechniqueORContinuous Renal Replacement ProcedureORContinuous RRTORContinuous RRTsORRRT, ContinuousORHemofiltration, Continuous Arteriovenous OR Arteriovenous Hemofiltration, ContinuousOR Continuous Arteriovenous HemofiltrationORContinuous Arteriovenous HemofiltrationsORContinuous Arterio-Venous Ultrafiltration OR Arterio-VenousUltrafiltration,Continuous ORContinuousArterioVenousUltrafiltration ORContinuous Arterio-VenousUltrafiltrations OR Ultrafiltration,ContinuousArterio-Venou sORCAVHDORContinuous Venovenous Hemodiafiltration ORContinuous Venovenous Hemodiafiltrations ORHemodiafiltration,ContinuousVenovenous OR Venovenous Hemodiafiltration,Continuous ORContinuousVeno-Venous Hemodiafiltration OR Continuous Veno Venous Hemodiafiltration[Title/Abstract]))) (("Plasma Exchange"[Mesh]) OR (Exchange, PlasmaORExchanges, PlasmaORPlasma Exchanges[Title/Abstract])) |
| --- | --- |
| Embase | ('severe acute pancreatitis'/exp OR 'severe acute pancreatitis' OR (severe AND acute AND ('pancreatitis'/exp OR pancreatitis))) AND hemofiltration  ('severe acute pancreatitis'/exp OR 'severe acute pancreatitis' OR (severe AND acute AND ('pancreatitis'/exp OR pancreatitis))) AND hemoperfusion  ('severe acute pancreatitis'/exp OR 'severe acute pancreatitis' OR (severe AND acute AND ('pancreatitis'/exp OR pancreatitis))) AND hemodialysis  ('severe acute pancreatitis'/exp OR 'severe acute pancreatitis' OR (severe AND acute AND ('pancreatitis'/exp OR pancreatitis))) AND hemodiafiltration  ('severe acute pancreatitis'/exp OR 'severe acute pancreatitis' OR (severe AND acute AND ('pancreatitis'/exp OR pancreatitis))) AND plasma exchange  ('severe acute pancreatitis'/exp OR 'severe acute pancreatitis' OR (severe AND acute AND ('pancreatitis'/exp OR pancreatitis))) AND crrt |
| web of science | **severe acute pancreatitis** (All Fields) and **hemofiltration** (Title)  **severe acute pancreatitis** (All Fields) and **hemoperfusion** (Title)  **severe acute pancreatitis** (All Fields) and **hemodialysis** (Title)  **severe acute pancreatitis** (All Fields) and **hemodiafiltration** (Title)  **severe acute pancreatitis** (All Fields) and plasma exchange(Title)  **severe acute pancreatitis** (All Fields) and CRRT(Title) |
| Cochrane Library | (severe acute pancreatitis):ti,ab,kw AND (**hemofiltration**)  (severe acute pancreatitis):ti,ab,kw AND (**hemoperfusion)**  (severe acute pancreatitis):ti,ab,kw AND (**hemodialysis)**  (severe acute pancreatitis):ti,ab,kw AND (**hemodiafiltration)**  (severe acute pancreatitis):ti,ab,kw AND (plasma exchange**)**  (severe acute pancreatitis):ti,ab,kw AND (CRRT) |
| CNKI | (SU=severe acute pancreatitis AND TI=hemofiltration)  OR(SU=severe acute pancreatitis AND TI=hemodialysis)  OR(SU=severe acute pancreatitis AND TI=plasma exchange)  OR(SU=severe acute pancreatitis AND TI=blood purification)  OR(SU=severe acute pancreatitis AND TI=CVVH)  OR(SU=severe acute pancreatitis AND TI=**hemodiafiltration**)  OR(SU=severe acute pancreatitis AND TI=hemoperfusion)  OR(SU=severe acute pancreatitis AND TI=CRRT)  OR(SU=severe acute pancreatitis AND TI=CRRT) |
| VIP | U=severe acute pancreatitis AND M=blood purification  U=severe acute pancreatitis AND M=hemodialysis  U=severe acute pancreatitis AND M=**hemodiafiltration**  U=severe acute pancreatitis AND M=plasma exchange  U=severe acute pancreatitis AND M=hemoperfusion  U=severe acute pancreatitis AND M= CRRT  U=severe acute pancreatitis AND M=CVVH  U=severe acute pancreatitis AND M=continuous renal replacement therapy |
| Wangfang Data | topic=severe acute pancreatitis AND Title/Abstract=blood purification  topic=severe acute pancreatitis AND Title/Abstract=hemodialysis  topic=severe acute pancreatitis AND Title/Abstract=hemofiltration  topic=severe acute pancreatitis AND Title/Abstract=**hemodiafiltration**  topic=severe acute pancreatitis AND Title/Abstract=plasma exchange  topic=severe acute pancreatitis AND Title/Abstract=hemoperfusion  topic=severe acute pancreatitis AND Title/Abstract=CRRT  topic=severe acute pancreatitis AND Title/Abstract=CVVH  topic=severe acute pancreatitis AND Title/Abstract=continuous renal replacement therapy |
| CBM | ( "blood purification"[Common field: intelligence] AND "severe acute pancreatitis"[Common field: intelligence])  ( "hemodialysis"[Common field: intelligence] AND "severe acute pancreatitis"[Common field: intelligence])  ( "**hemodiafiltration**"[Common field: intelligence] AND "severe acute pancreatitis"[Common field: intelligence])  ( "plasma exchange"[Common field: intelligence] AND "severe acute pancreatitis"[Common field: intelligence])  ( "hemoperfusion"[Common field: intelligence] AND "severe acute pancreatitis"[Common field: intelligence])  ( "CRRT "[Common field: intelligence] AND "severe acute pancreatitis"[Common field: intelligence])  ( "CVVH"[Common field: intelligence] AND "severe acute pancreatitis"[Common field: intelligence])  ( "continuous renal replacement therapy "[Common field: intelligence] AND "severe acute pancreatitis"[Common field: intelligence]) |

**Supplementary Figure 1**. Change from baseline calculation formula.

*M _change_ = M _final_ −M _baseline_*


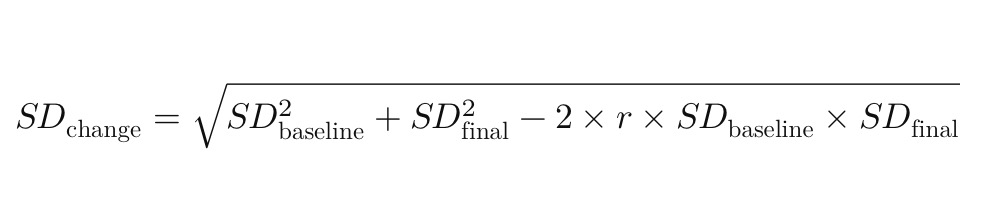


*M _change_* = mean change from baseline

*M _final_* = mean value at the end of intervention

*M _baseline_* = mean value at baseline

*SD _change_* = standard deviation of the change

*SD _baseline_* = standard deviation at baseline

*SD _final_* = standard deviation at the end of the intervention

*r* = correlation coefficient between baseline and final measurements, usually choose 0.5

**Supplementary Table 2**. Characteristics of the trials included in network meta-analysis.

| **Number** | **Author + Year** | **Random method** | **Intervention measures** | | **Sample size** | | **Male/Female/Example** | | | | **Age** | | **Intervention time/d** |
| --- | --- | --- | --- | --- | --- | --- | --- | --- | --- | --- | --- | --- | --- |
|  |  |  | **Control group** | **Experimental group** | **Control group** | **Experimental group** | **Control group♂** | **Control group♀** | **Experimental group♂** | **Experimental group♀** | **Control group** | **Experimental group** |  |
| 1 | Tang2016^[15]^ | Digital Notation | PT | L | 47 | 48 | 25 | 22 | 26 | 22 | 51.39±8.52 | 50.28±8.12 | 5 |
| 2 | Peng2019^[16]^ | Digital Notation | PT | C | 41 | 41 | 16 | 11 | 17 | 10 | 44.3±8.50 | 43.7±8.6 | / |
| 3 | Hu2020^[17]^ | Digital Notation | PT | C/H+C | 45 | 45/45 | 19 | 26/21 | 21 | 19/24 | 34.71±2.81 | 34.71±2.81/34.56±2.79 | 3 |
| 4 | Yin2020^[18]^ | Computer Random Selection | T | C | 30 | 30 | 13 | 17 | 12 | 18 | 38.26±4.4 | 35.35±4.3 | / |
| 5 | Liao2017^[19]^ | Digital Notation | PT | H+T | 38 | 38 | 19 | 19 | 18 | 20 | 39.5±6.4 | 38.9±6.1 | / |
| 6 | Ye2019^[20]^ | Digital Notation | C | H+C | 43 | 43 | 25 | 18 | 23 | 20 | 41.98±9.24 | 53.16±8.92 | 7 |
| 7 | Xie2019^[21]^ | Hospitalization Number | C | C+PE | 69 | 66 | 39 | 31 | 37 | 29 | 48.6±1.8 | 50.1±1.9 | / |
| 8 | Li2016^[22]^ | Random | T | H+T | 46 | 46 | 53 | 39 | 53 | 39 | / | / | 50.5±8.5h |
| 9 | Dan2020^[23]^ | Random | PT | C | 41 | 42 | 28 | 13 | 30 | 12 | 60.12±5.47 | 60.15±5.62 | / |
| 10 | Zhou2019^[24]^ | Digital Notation | PT | H+C | 31 | 31 | 17 | 14 | 18 | 13 | 52.32±2.20 | 52.48±2.3 | 3 |
| 11 | Chen2021^[25]^ | Random | PT | H+C | 32 | 32 | 17 | 15 | 18 | 15 | 48.64±7.63 | 48.87±7.84 | 3 |
| 12 | Zhang2021^[26]^ | Computer Random Selection | PT | H+C | 41 | 41 | 26 | 15 | 27 | 14 | 37.42±2.83 | 36.59±2.77 | 7 |
| 13 | Cui2021^[27]^ | Digital Notation | PT | H+L | 38 | 38 | 20 | 18 | 21 | 17 | 55.8±6.9 | 56.3±7.2 | 3 |
| 14 | Huang2024^[28]^ | Digital Notation | PT | H+C | 54 | 54 | 31 | 23 | 28 | 26 | 47.6±6.4 | 47.3±6.5 | / |
| 15 | Wu2022^[29]^ | Digital Notation | PT | C | 50 | 50 | 26 | 24 | 28 | 22 | 63.48±9.2 | 62.7±8.19 | 3 |
| 16 | Maihemuti2018^[30]^ | Digital Notation | PT | C | 74 | 74 | 41 | 33 | 39 | 35 | 42.35±3.23 | 43.75±4.12 | 28 |
| 17 | Gan2024^[31]^ | Digital Notation | C | H+C | 34 | 34 | 21 | 13 | 20 | 14 | 45.27±5.86 | 45.26±5.81 | 7 |
| 18 | Li2021^[32]^ | Double Color Ball | PT | L | 33 | 33 | 19 | 14 | 18 | 15 | 45.11±1.11 | 45.12±1.12 | 14 |
| 19 | Xie2018^[33]^ | Digital Notation | PT | C | 34 | 34 | 18 | 16 | 17 | 17 | 57.68±12.87 | 58.67±7.66 | 7 |
| 20 | Shi2022^[34]^ | Digital Notation | PT | C | 39 | 39 | 20 | 19 | 21 | 18 | 48.18±55.89 | 48.76±5.13 | 7 |
| 21 | He2021^[35]^ | Digital Notation | C | H+C | 39 | 39 | 26 | 13 | 25 | 14 | 51.64±6.53 | 51.82±6.03 | 5 |
| 22 | Huang2023^[36]^ | Digital Notation | PT | H+C | 25 | 25 | 13 | 12 | 15 | 10 | 42.83±1.62 | 42.51±1.83 | / |
| 23 | Zeng2020^[37]^ | Digital Notation | PT | C | 36 | 36 | 19 | 17 | 21 | 15 | 52.26±9.81 | 53.59±9.58 | 3 |
| 24 | Qiu2018^[38]^ | Random | PT | L | 49 | 49 | 28 | 21 | 25 | 24 | 54.23±13.45 | 55.21±14.35 | / |
| 25 | Chen2017^[39]^ | Digital Notation | PT | C | 30 | 30 | 17 | 13 | 18 | 12 | 46.1±13.7 | 47.2±11.3 | 7 |
| 26 | [Xu2022](https://kns.cnki.net/kcms2/author/detail?v=UQND20kjH6Iy24X9pgIYYEDKmh7moAUfDImpXD-FhfeafHE1nvxFWNrAmRHIMpOmIsFVzUGH8gXMm1VKYfYkwT4Xx1wfyxlO-2OEBigAn5L5wQWW6K5DaY-oCnVf4oxz&uniplatform=NZKPT&language=CHS)^[40]^ | Digital Notation | C | C+PE | 15 | 15 | 8 | 7 | 9 | 6 | 43.75±8.37 | 43.96±8.45 | / |
| 27 | Pu2017^[41]^ | Random | PT | PE+T+L | 100 | 100 | 46 | 54 | 47 | 53 | 24-60 | 25-60 |  |
| 28 | Lin2018^[42]^ | Random | PT | T+L | 32 | 32 | 19 | 13 | 20 | 12 | 56.7±7.3 | 57±7.5 | / |
| 29 | Duan2020^[43]^ | Digital Notation | PT | L | 33 | 34 | 18 | 15 | 17 | 17 | 45.96±7.12 | 45.75±7.14 | 7 |
| 30 | Wu2015^[44]^ | Random | PT | PE | 28 | 28 | 20 | 8 | 18 | 10 | 40.8±28.1 | 42.3±26.5 | 5 |
| 31 | [Ding2021](https://kns.cnki.net/kcms2/author/detail?v=UQND20kjH6LrCb2Ra-DVweLou5Rgy4aXrbKQatrRYvksv4PLFooue7DJ7ZIUTXdR01y0rsijO5aLaiGl008QXJDBE3GF3q3ZLLn5nH1ZYpNCKTIqoHj4tfkQENsjdi4d&uniplatform=NZKPT&language=CHS)^[45]^ | Random | C | C+PE | 49 | 49 | 27 | 22 | 26 | 23 | 57.38±10.45 | 55.5±9.4 | 3 |
| 32 | Zeng2024^[46]^ | Digital Notation | C | H+C | 42 | 43 | 24 | 18 | 26 | 17 | 39.13±10.28 | 40.53±11.25 | 2 |
| 33 | Liu2019^[47]^ | Digital Notation | PT | H | 40 | 40 | 22 | 18 | 23 | 17 | 42.85±6.18 | 42.57±6.12 | 7 |
| 34 | Pan2019^[48]^ | Digital Notation | C | H+C | 40 | 40 | 22 | 18 | 24 | 16 | 40.38±7.14 | 41.69±7.53 | 3 |
| 35 | Luo2015^[19]^ | Random | PT | C | 56 | 52 | 39 | 17 | 36 | 16 | 54.3±10.53 | 53.32±10.81 | 5 |
| 36 | Jin2018^[50]^ | Digital Notation | T+L | H+T+L | 44 | 44 | 19 | 25 | 21 | 23 | 41.51±10.52 | 39.73±12.3 | 14 |
| 37 | Zhu2016^[51]^ | Random | PT | H+C | 27 | 27 | 16 | 11 | 15 | 12 | 34-56 | 36-55 | 3 |
| 38 | Lu2022^[52]^ | Digital Notation | PT | H+C | 30 | 30 | 21 | 9 | 23 | 7 | 42.1±11.63 | 41.56±12.92 | 5 |
| 39 | Liu2020^[53]^ | Random | PT | C | 33 | 33 | 19 | 14 | 18 | 15 | 45.5±10 | 46±10.5 | 5 |
| 40 | Mi2018^[54]^ | Random | C | H+C | 33 | 33 | 18 | 15 | 20 | 13 | 43.64±4.8 | 5-31h | 14 |
| 41 | Huang2022^[55]^ | Digital Notation | PT | C | 82 | 82 | 39 | 43 | 40 | 42 | 38.72±13.26 | 37.81±13.26 | 7 |
| 42 | Guo2016^[56]^ | Random | PT | C | 32 | 32 | / | / | / | / | 51.58±12.64 | 52.31±11.96 | 3 |
| 43 | [Guo2014](https://pubmed.ncbi.nlm.nih.gov/?sort=pubdate&size=200&term=Guo+J&cauthor_id=24518504)^[57]^ | Random | PT | C | 29 | 32 | 10 | 19 | 12 | 20 | 48.2±12.5 | 53±15.7 | 7 |
| 44 | Sun2015^[58]^ | Not mentioned | PT | H+C | 10 | 10 | 8 | 2 | 7 | 3 | 40.25±1.63 | 43.2±1.03 | 2 |
| 45 | [Xie2021](https://pubmed.ncbi.nlm.nih.gov/?sort=pubdate&size=200&term=Xie+Y&cauthor_id=34871235)^[59]^ | Random | PT | C | 15/16 | 2122 | 11/10 | 4/6 | 12/17 | 9/5 | 47.2±8.5/45.6±7.4 | 50.2±9.2/46.8±8.6 | 7 |

*Continuous renal replacement therapy (C), Continuous renal replacement therapy + Plasma exchange (C+PE), Hemodialysis (HD), Hemodiafiltration (HDF), Hemofiltration (HF), Hemoperfusion + Continuous renal replacement therapy (HP+C), Hemoperfusion + Hemodialysis (HP+HD), Hemoperfusion + Hemodiafiltration (HP+HDF), Non-blood purification group (N), Plasma exchange (PE), Plasma exchange + Hemodialysis (PE+HD)*

**Supplementary table 3.** Inconsistency test P value

|  | AMS | CRP | IL-6 | Scr | APACHEⅡ | TG | Death Toll | Ineffectivenss |
| --- | --- | --- | --- | --- | --- | --- | --- | --- |
| P | 0.3907 | 0.9152 | 0.3755 | 0.4036 | 0.887 | 0.7504 | 0.3187 | 0.1994 |

*P>0.05 indicates that the inconsistency is not significant*

**Supplementary Figure 2.** Node-splitting analyses result

1.AMS


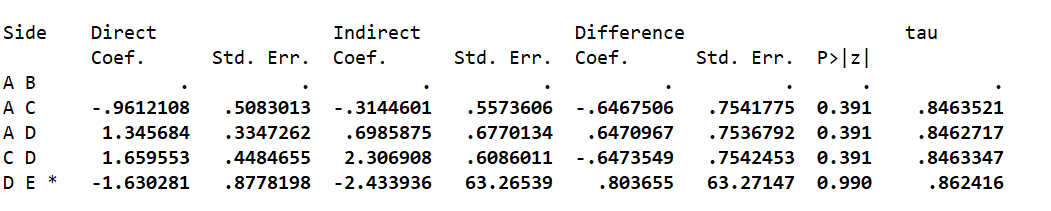


AMS after excluding high-risk studies


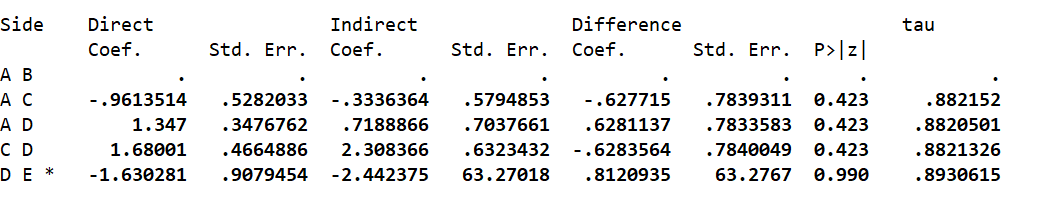


2.CRP


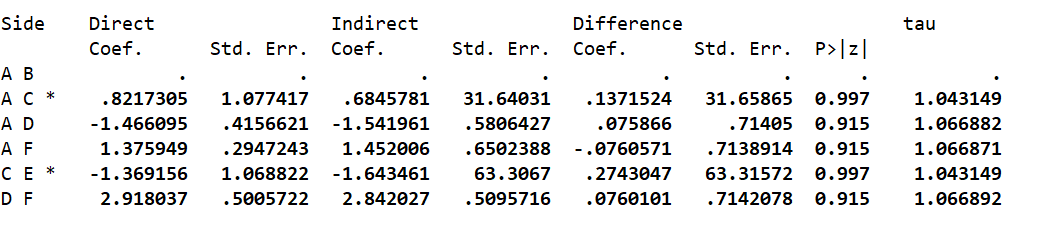


CRP after excluding high-risk studies


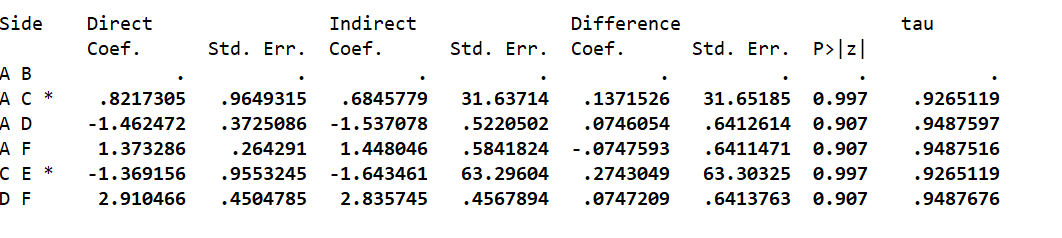


1. IL-6


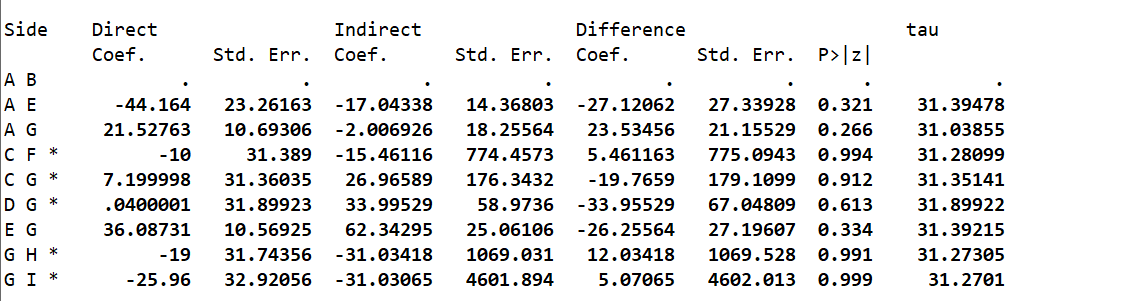


4.Scr


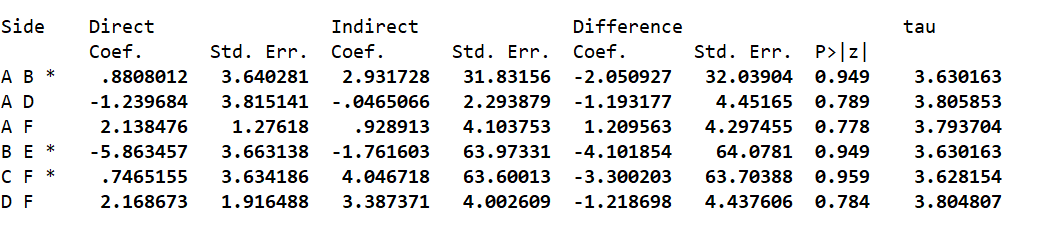


5.APACHEⅡ


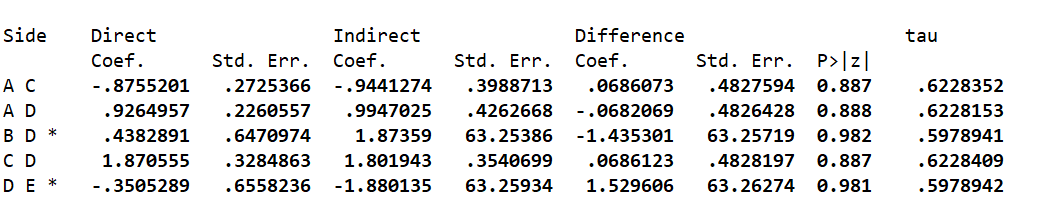


6.TG


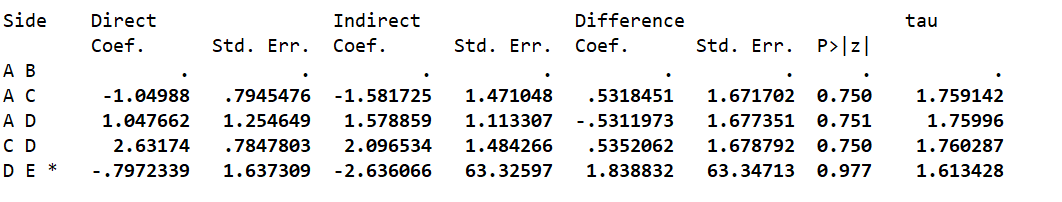


7.mortality


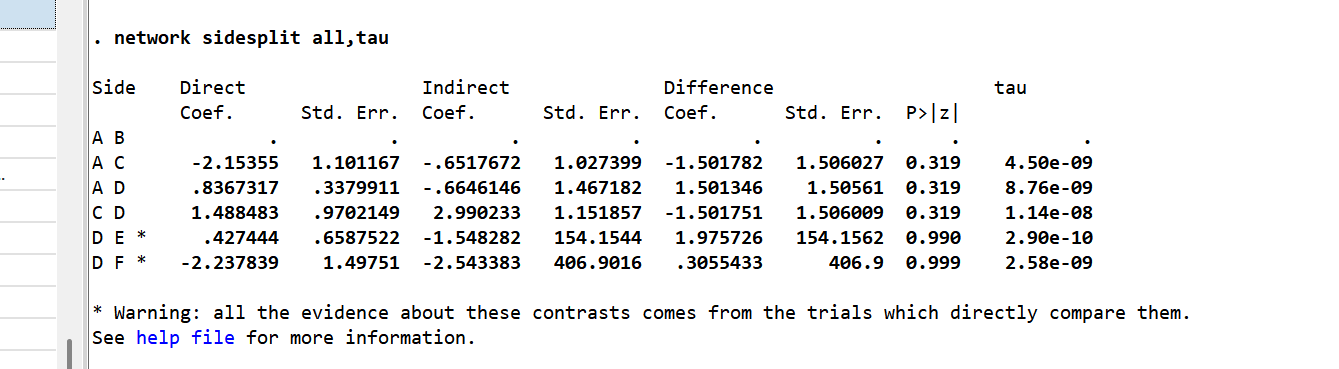


*P>0.05 indicates no significant inconsistency locally*

**Supplementary Table 4**.Comparative effect of different blood purification modes in improving AMS Scores in SAP Patients(MD,95% CI) Significant results are Highlighted in red.

|  | **PE+HD** | **N** | **HP+C** | **C+PE** | **C** |
| --- | --- | --- | --- | --- | --- |
| **PE+HD** | 0 |  |  |  |  |
| **N** | -1.63 (-3.32,0.06) | 0 |  |  |  |
| **HP+C** | 0.68 (-1.39,2.74) | 2.31 (1.11,3.50) | 0 |  |  |
| **C+PE** | 0.53 (-1.54,2.60) | 2.16 (0.96,3.36) | -0.14 (-1.56,1.27) | 0 |  |
| **C** | 1.35 (0.69,2.00) | -0.65 (-2.12,0.83) | -0.96 (-1.96,0.03) | -0.82 (-1.82,0.19) | 0 |

*Continuous renal replacement therapy (C), Continuous renal replacement therapy + Plasma exchange (C+PE), Hemodialysis (HD), Hemodiafiltration (HDF), Hemofiltration (HF), Hemoperfusion + Continuous renal replacement therapy (HP+C), Hemoperfusion + Hemodialysis (HP+HD), Hemoperfusion + Hemodiafiltration (HP+HDF), Non-blood purification group (N), Plasma exchange (PE), Plasma exchange + Hemodialysis (PE+HD)*

**Supplementary Table 5**. Comparative effect of different blood purification modes in improving AMS scores in SAP patients after excluding study at high risk of bias (MD,95% CI). Significant results are highlighted in red.

|  | PE+HD | N | HP+C | C+PE | C |
| --- | --- | --- | --- | --- | --- |
| **PE+HD** | 0 |  |  |  |  |
| **N** | -1.63 (-3.39,0.13) | 0 |  |  |  |
| **HP+C** | 0.68 (-1.47,2.83) | 2.31 (1.07,3.55) | 0 |  |  |
| **C+PE** | 0.78 (-1.50,3.07) | 2.41 (0.95,3.88) | 0.11 (-1.55,1.76) | 0 |  |
| **C** | 1.35 (0.67,2.03) | -0.63 (-2.16,0.91) | -0.96 (-2.00,0.07) | -1.07 (-2.36,0.23) | 0 |

*Continuous renal replacement therapy (C), Continuous renal replacement therapy + Plasma exchange (C+PE), Hemodialysis (HD), Hemodiafiltration (HDF), Hemofiltration (HF), Hemoperfusion + Continuous renal replacement therapy (HP+C), Hemoperfusion + Hemodialysis (HP+HD), Hemoperfusion + Hemodiafiltration (HP+HDF), Non-blood purification group (N), Plasma exchange (PE), Plasma exchange + Hemodialysis (PE+HD)*

**Supplementary Table 6**.Comparative effect of different blood purification modes in improving CRP Scores in SAP Patients(SMD,95% CI) Significant results are Highlighted in red.

|  | N | HP+HD | HP+C | HD | C+PE | C |
| --- | --- | --- | --- | --- | --- | --- |
| N | 0 |  |  |  |  |  |
| HP+HD | 1.94 (-1.08,4.95) | 0 |  |  |  |  |
| HP+C | 2.88 (2.19,3.57) | 0.94 (-2.10,3.99) | 0 |  |  |  |
| HD | 0.57 (-1.61,2.74) | -1.37 (-3.46,0.73) | -2.31 (-4.52,-0.10) | 0 |  |  |
| C+PE | 4.30 (2.68,5.91) | 2.36 (-0.98,5.70) | 1.42 (-0.25,3.08) | 3.73 (1.12,6.34) | 0 |  |
| C | 1.39 (0.87,1.90) | -0.55 (-3.52,2.42) | -1.49 (-2.14,-0.84) | 0.82 (-1.29,2.93) | -2.91 (-4.44,-1.38) | 0 |

*Continuous renal replacement therapy (C), Continuous renal replacement therapy + Plasma exchange (C+PE), Hemodialysis (HD), Hemodiafiltration (HDF), Hemofiltration (HF), Hemoperfusion + Continuous renal replacement therapy (HP+C), Hemoperfusion + Hemodialysis (HP+HD), Hemoperfusion + Hemodiafiltration (HP+HDF), Non-blood purification group (N), Plasma exchange (PE), Plasma exchange + Hemodialysis (PE+HD)*

**Supplementary Table 7**. Comparative effect of different blood purification modes in improving CRP scores in SAP patients after excluding study at high risk of bias (SMD,95% CI). Significant results are highlighted in red.

|  | N | HP+HD | HP+C | HD | C+PE | C |
| --- | --- | --- | --- | --- | --- | --- |
| N | 0 |  |  |  |  |  |
| HP+HD | 1.93 (-0.77,4.63) | 0 |  |  |  |  |
| HP+C | 2.87 (2.26,3.49) | 0.94 (-1.78,3.66) | 0 |  |  |  |
| HD | 0.56 (-1.38,2.51) | -1.37 (-3.24,0.50) | -2.31 (-4.29,-0.33) | 0 |  |  |
| C+PE | 2.39 (0.37,4.41) | 0.46 (-2.85,3.77) | -0.48 (-2.54,1.57) | 1.83 (-0.90,4.56) | 0 |  |
| C | 1.39 (0.92,1.85) | -0.55 (-3.21,2.11) | -1.49 (-2.07,-0.91) | 0.82 (-1.07,2.71) | -1.01 (-2.98,0.97) | 0 |

*Continuous renal replacement therapy (C), Continuous renal replacement therapy + Plasma exchange (C+PE), Hemodialysis (HD), Hemodiafiltration (HDF), Hemofiltration (HF), Hemoperfusion + Continuous renal replacement therapy (HP+C), Hemoperfusion + Hemodialysis (HP+HD), Hemoperfusion + Hemodiafiltration (HP+HDF), Non-blood purification group (N), Plasma exchange (PE), Plasma exchange + Hemodialysis (PE+HD)*

**Supplementary Table 8**.Comparative effect of different blood purification modes in improving IL-6 Scores in SAP Patients(MD,95% CI) Significant results are Highlighted in red.

|  | PE+HD | PE | N | HP+HDF | HP+C | HF | HDF | C+PE | C |
| --- | --- | --- | --- | --- | --- | --- | --- | --- | --- |
| PE+HD | 0 |  |  |  |  |  |  |  |  |
| PE | 0.56 (-3.09,4.21) | 0 |  |  |  |  |  |  |  |
| N | -0.36 (-2.91,2.20) | -0.92 (-3.52,1.69) | 0 |  |  |  |  |  |  |
| HP+HDF | 2.83 (-1.65,7.31) | 2.26 (-2.24,6.77) | 3.18 (-0.50,6.86) | 0 |  |  |  |  |  |
| HP+C | 1.79 (-0.89,4.47) | 1.23 (-1.50,3.95) | 2.14 (1.34,2.95) | -1.04 (-4.80,2.73) | 0 |  |  |  |  |
| HF | 0.08 (-3.56,3.72) | -0.48 (-4.15,3.19) | 0.44 (-2.15,3.03) | -2.75 (-7.24,1.75) | -1.71 (-4.42,1.00) | 0 |  |  |  |
| HDF | 2.01 (-1.65,5.68) | 1.45 (-2.24,5.15) | 2.37 (-0.25,4.99) | -0.81 (-3.39,1.77) | 0.23 (-2.52,2.97) | 1.93 (-1.75,5.62) | 0 |  |  |
| C+PE | 2.98 (-0.30,6.26) | 2.41 (-0.90,5.73) | 3.33 (1.28,5.38) | 0.15 (-4.06,4.36) | 1.19 (-0.96,3.34) | 2.90 (-0.41,6.20) | 0.96 (-2.37,4.29) | 0 |  |
| C | 0.94 (-1.74,3.62) | 0.38 (-2.34,3.10) | 1.30 (0.50,2.09) | -1.89 (-5.65,1.88) | -0.85 (-1.87,0.17) | 0.86 (-1.85,3.57) | -1.08 (-3.82,1.67) | -2.04 (-3.93,-0.15) | 0 |

*Continuous renal replacement therapy (C), Continuous renal replacement therapy + Plasma exchange (C+PE), Hemodialysis (HD), Hemodiafiltration (HDF), Hemofiltration (HF), Hemoperfusion + Continuous renal replacement therapy (HP+C), Hemoperfusion + Hemodialysis (HP+HD), Hemoperfusion + Hemodiafiltration (HP+HDF), Non-blood purification group (N), Plasma exchange (PE), Plasma exchange + Hemodialysis (PE+HD)*

**Supplementary Table 9**. Comparative effect of different blood purification modes in improving Scr Scores in SAP Patients(MD,95% CI) Significant results are Highlighted in red.

|  | **N** | **HP+HD** | **HP+C** | **HF** | **HD** | **C** |
| --- | --- | --- | --- | --- | --- | --- |
| **N** | 0 |  |  |  |  |  |
| **HP+HD** | 100.69 (-80.00,281.38) | 0 |  |  |  |  |
| **HP+C** | 136.18 (6.27,266.09) | 35.50 (-179.27,250.26) | 0 |  |  |  |
| **HF** | 37.59 (-84.44,159.61) | -63.10 (-281.13,154.93) | -98.60 (-276.82,79.63) | 0 |  |  |
| **HD** | -29.18 (-164.16,105.80) | -129.87 (-250.52,-9.21) | -165.36 (-343.40,12.68) | -66.77 (-248.72,115.19) | 0 |  |
| **C** | -61.82 (-206.89,83.25) | -48.02 (-223.94,127.90) | -83.51 (-206.71,39.69) | 15.08 (-113.71,143.88) | 81.85 (-46.68,210.38) | 0 |

*Continuous renal replacement therapy (C), Continuous renal replacement therapy + Plasma exchange (C+PE), Hemodialysis (HD), Hemodiafiltration (HDF), Hemofiltration (HF), Hemoperfusion + Continuous renal replacement therapy (HP+C), Hemoperfusion + Hemodialysis (HP+HD), Hemoperfusion + Hemodiafiltration (HP+HDF), Non-blood purification group (N), Plasma exchange (PE), Plasma exchange + Hemodialysis (PE+HD)*

**Supplementary Table 10**.Comparative effect of different blood purification modes in improving APACHE II Scores in SAP (MD,95% CI). Significant results are highlighted in red.

|  | **PE** | **N** | **HP+C** | **HF** | **C** |
| --- | --- | --- | --- | --- | --- |
| **PE** | 0 |  |  |  |  |
| **N** | -0.35 (-1.64,0.93) | 0 |  |  |  |
| **HP+C** | 1.48 (0.12,2.85) | 1.83 (1.38,2.29) | 0 |  |  |
| **HF** | 0.09 (-1.72,1.89) | 0.44 (-0.83,1.71) | -1.40 (-2.74,-0.05) | 0 |  |
| **C** | 0.59 (-0.75,1.93) | 0.94 (0.56,1.32) | -0.89 (-1.32,-0.47) | 0.50 (-0.82,1.82) | 0 |

*Continuous renal replacement therapy (C), Continuous renal replacement therapy + Plasma exchange (C+PE), Hemodialysis (HD), Hemodiafiltration (HDF), Hemofiltration (HF), Hemoperfusion + Continuous renal replacement therapy (HP+C), Hemoperfusion + Hemodialysis (HP+HD), Hemoperfusion + Hemodiafiltration (HP+HDF), Non-blood purification group (N), Plasma exchange (PE), Plasma exchange + Hemodialysis (PE+HD)*

**Supplementary Table 11**. Comparative effect of different blood purification modes in improving TG in Hyperlipidemic Severe Acute Pancreatitis Patients(MD,95% CI) . Significant results are highlighted in red.

|  | **PE** | **N** | **HP+C** | **C+PE** | **C** |
| --- | --- | --- | --- | --- | --- |
| **PE** | 0 |  |  |  |  |
| **N** | -0.80 (-4.01,2.41) | 0 |  |  |  |
| **HP+C** | 1.68 (-1.77,5.12) | 2.48 (1.22,3.74) | 0 |  |  |
| **C+PE** | 1.39 (-3.42,6.20) | 2.19 (-1.39,5.77) | -0.29 (-3.77,3.20) | 0 |  |
| **C** | 0.52 (-3.02,4.06) | 1.32 (-0.19,2.82) | -1.16 (-2.42,0.10) | -0.87 (-4.12,2.38) | 0 |

*Continuous renal replacement therapy (C), Continuous renal replacement therapy + Plasma exchange (C+PE), Hemodialysis (HD), Hemodiafiltration (HDF), Hemofiltration (HF), Hemoperfusion + Continuous renal replacement therapy (HP+C), Hemoperfusion + Hemodialysis (HP+HD), Hemoperfusion + Hemodiafiltration (HP+HDF), Non-blood purification group (N), Plasma exchange (PE), Plasma exchange + Hemodialysis (PE+HD)*

**Supplementary Table 12**.Comparative effect of different blood purification modes in reducing the Mortality in SAP Patients(RR,95% CI) Significant results are Highlighted in red.

|  | **PE+HD** | **PE** | **N** | **HP+C** | **HD** | **C** |
| --- | --- | --- | --- | --- | --- | --- |
| **PE+HD** | 0 |  |  |  |  |  |
| **PE** | 0.07 (0.00,1.72) | 0 |  |  |  |  |
| **N** | 0.11 (0.01,2.01) | 1.54 (0.42,5.58) | 0 |  |  |  |
| **HP+C** | 0.88 (0.03,23.34) | 12.68 (1.80,88.23) | 8.25 (1.93,35.52) | 0 |  |  |
| **HD** | 0.03 (0.00,0.85) | 0.41 (0.05,3.53) | 0.26 (0.05,1.51) | 0.03 (0.00,0.28) | 0 |  |
| **C** | 0.23 (0.01,4.62) | 3.29 (0.77,13.87) | 2.14 (1.13,4.10) | 0.26 (0.06,1.13) | 8.08 (1.62,40.85) | 0 |

*Continuous renal replacement therapy (C), Continuous renal replacement therapy + Plasma exchange (C+PE), Hemodialysis (HD), Hemodiafiltration (HDF), Hemofiltration (HF), Hemoperfusion + Continuous renal replacement therapy (HP+C), Hemoperfusion + Hemodialysis (HP+HD), Hemoperfusion + Hemodiafiltration (HP+HDF), Non-blood purification group (N), Plasma exchange (PE), Plasma exchange + Hemodialysis (PE+HD)*

**Supplementary Figure 3.** Meta analysis of Hemoperfusion combined with CRRT versus CRRT in AMS.


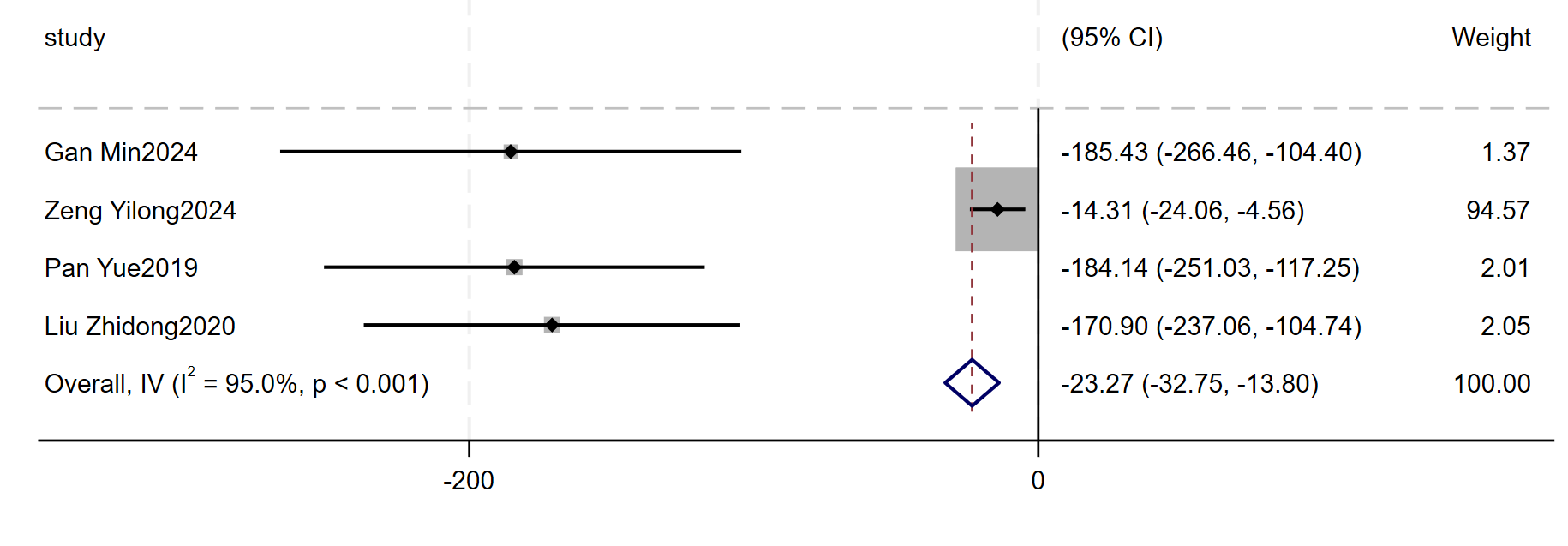


**Supplementary Figure 4.** Meta analysis of Hemoperfusion combined with CRRT versus CRRT in AMS after excluding the study by Zeng Yilong.

5
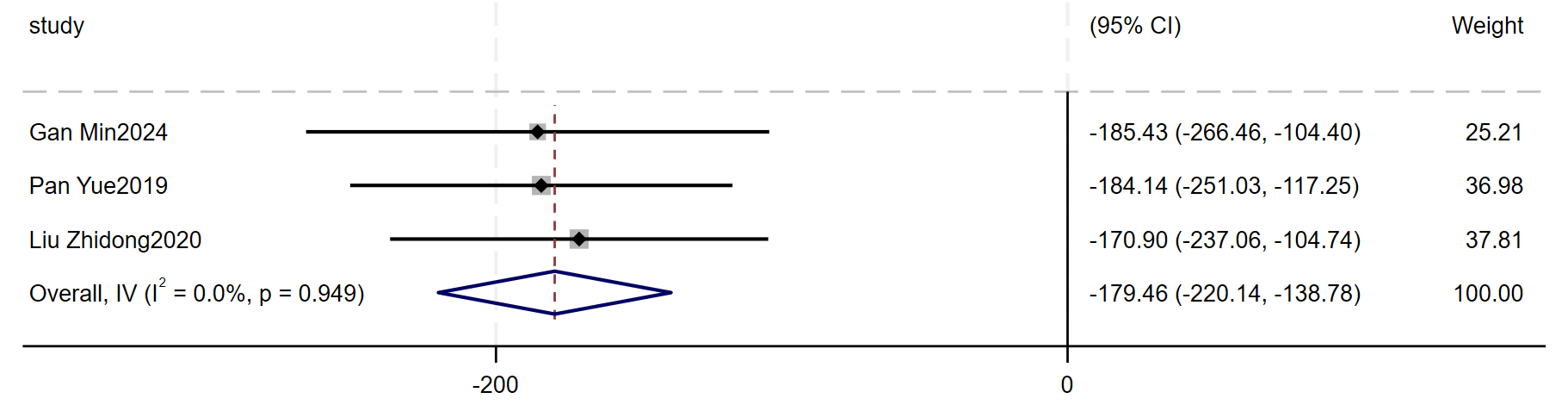


**Supplementary Figure 5.** Meta analysis of Hemoperfusion combined with CRRT versus Non-blood purification group in AMS.


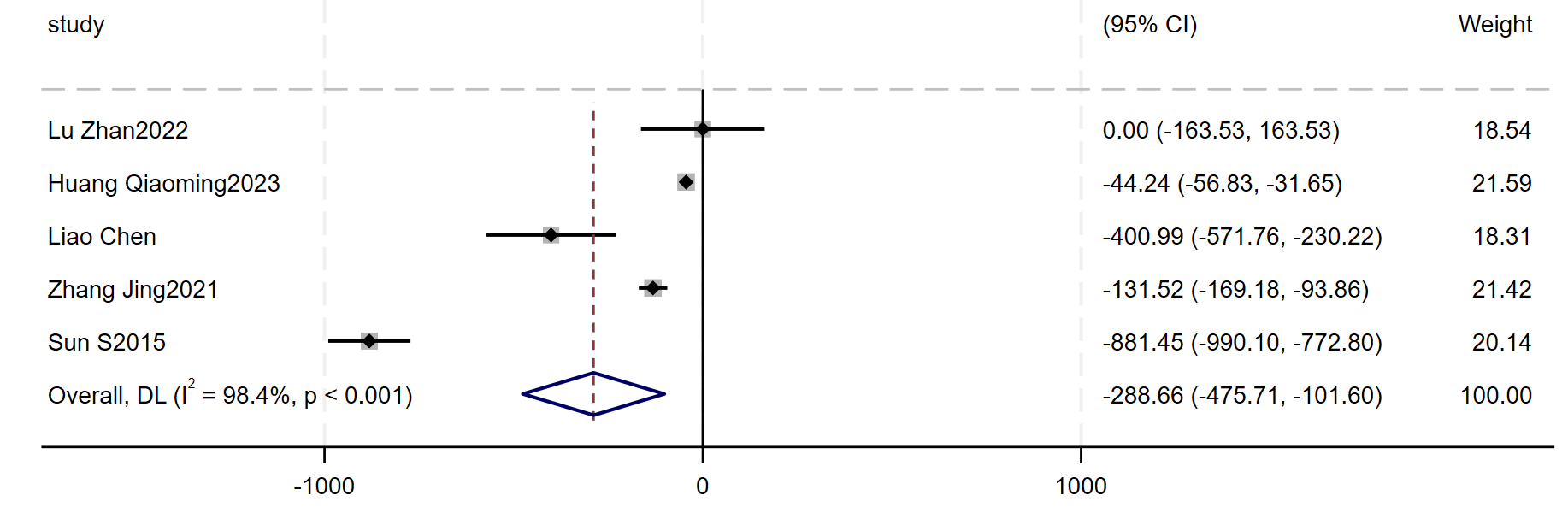


**Supplementary Figure 6.** Meta analysis of Hemoperfusion combined with CRRT versus Non-blood purification group in AMS after excluding the study by Sun S.


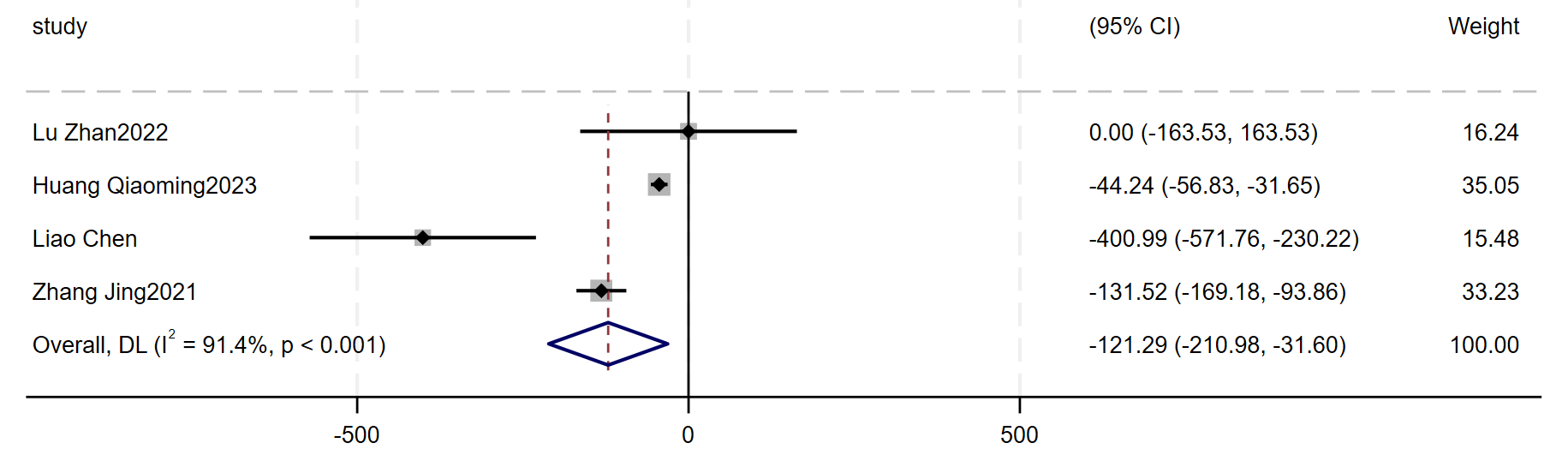


**Supplementary Figure 7.** Meta analysis of CRRT versus Non-blood purification group in AMS.


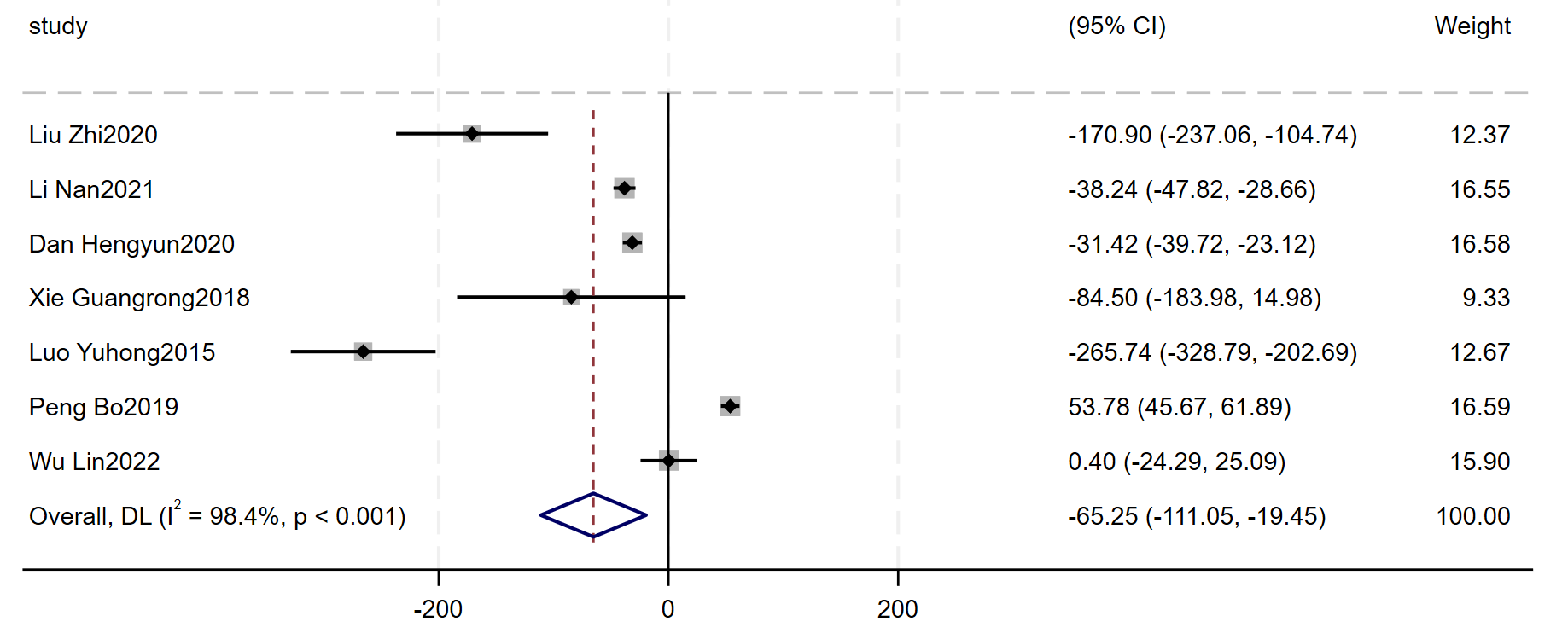


**Supplementary Figure 8.** Meta analysis of CRRT versus Non-blood purification group in AMS after excluding the study by Peng Bo.


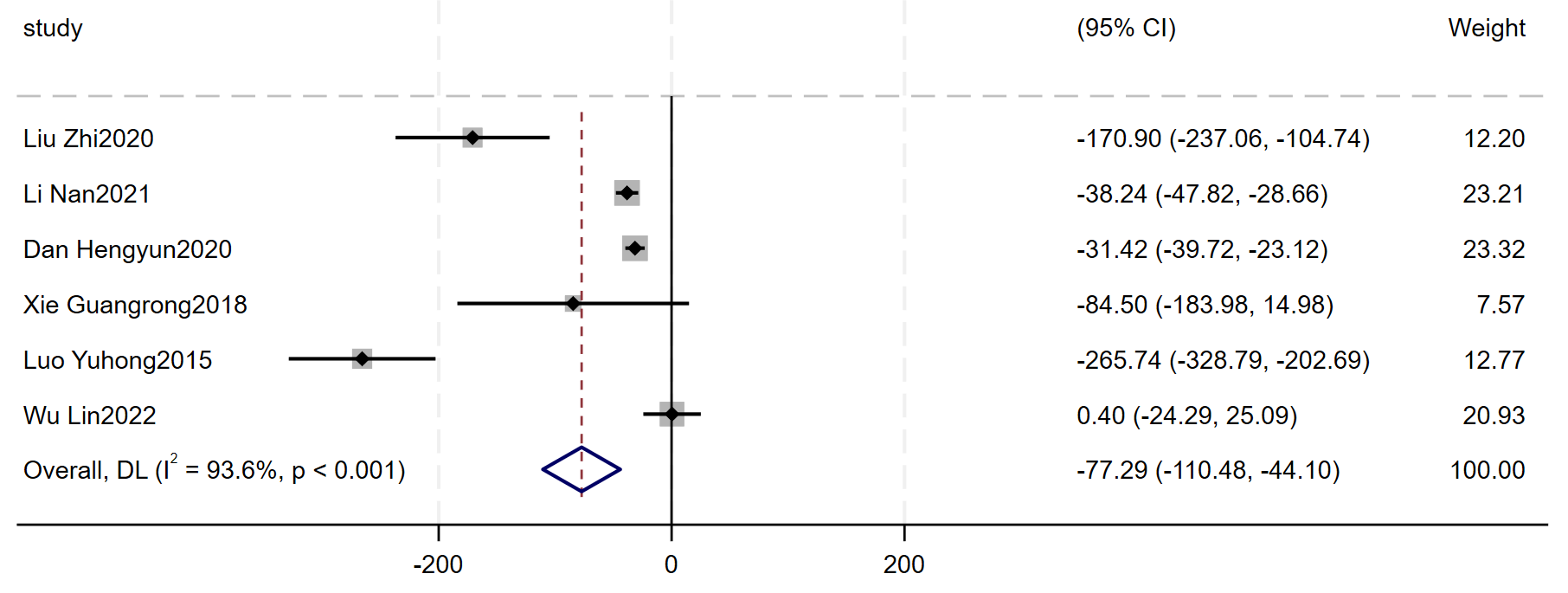


**Supplementary Figure 9.** Meta analysis of CRRT plus Plasma exchange versus CRRT in AMS before and after excluding the study by Xie Peipei.


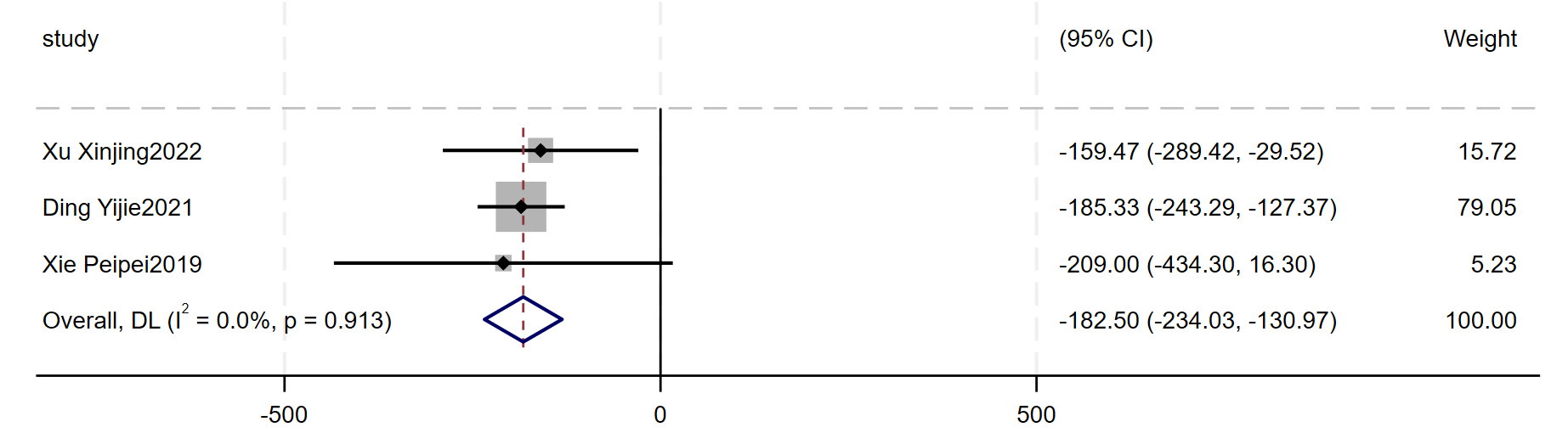


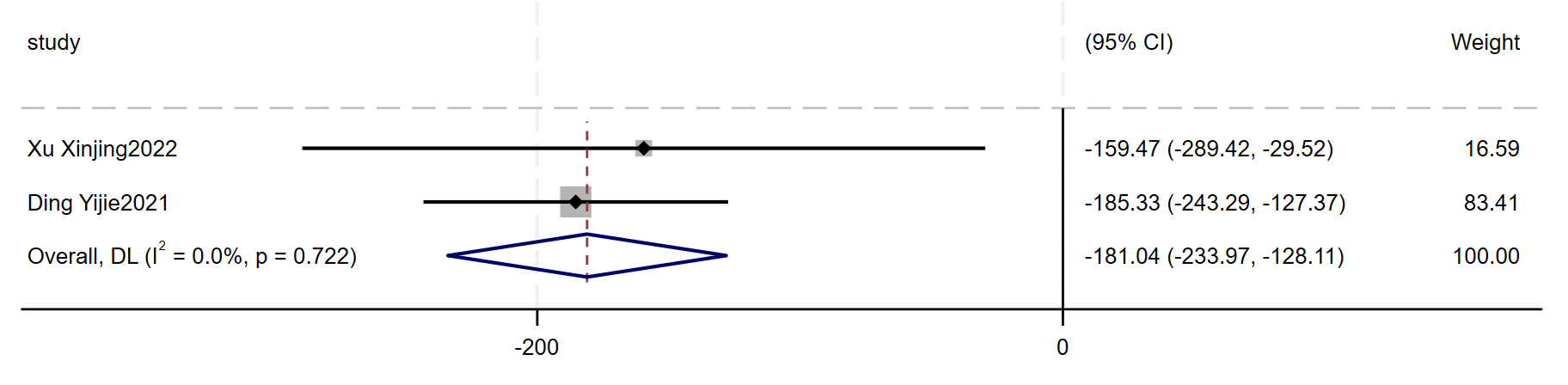


**Supplementary Figure 10.** Meta analysis of Hemoperfusion combined with CRRT versus CRRT in IL-6.


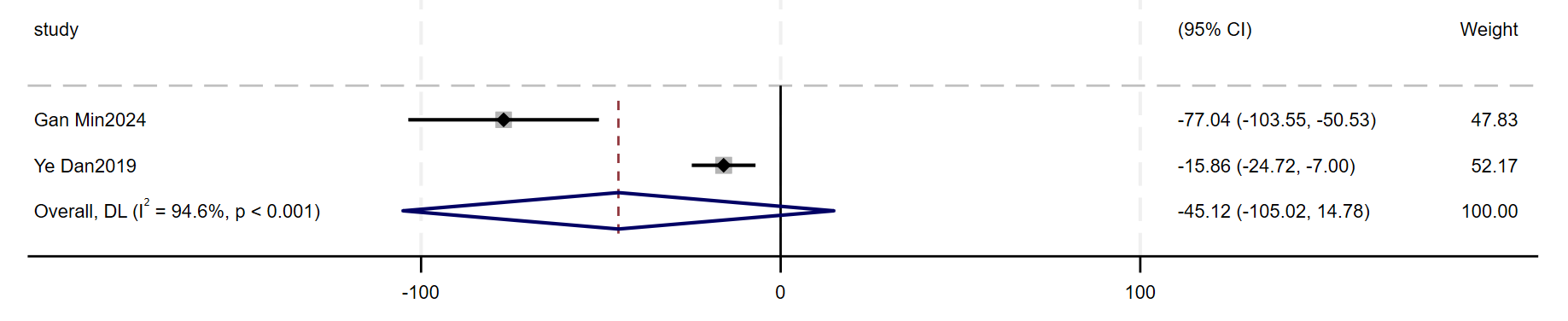


**Supplementary Figure 11.** Meta analysis of Hemoperfusion combined with CRRT versus Non-blood purification group in IL-6.


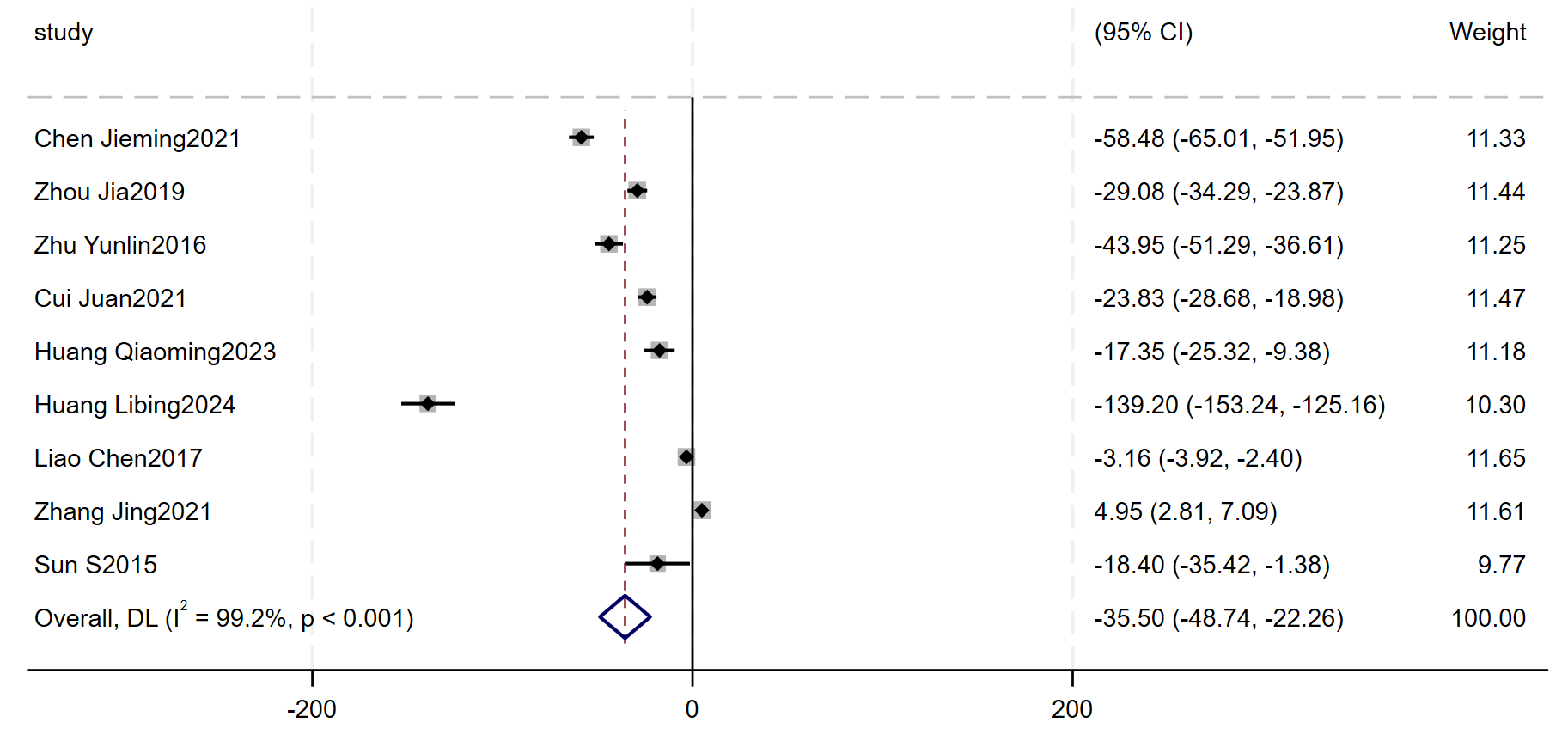


**Supplementary Figure 12.** Meta analysis of Hemoperfusion combined with CRRT versus Non-blood purification group in IL-6 after excluding the study by Huang Libing.


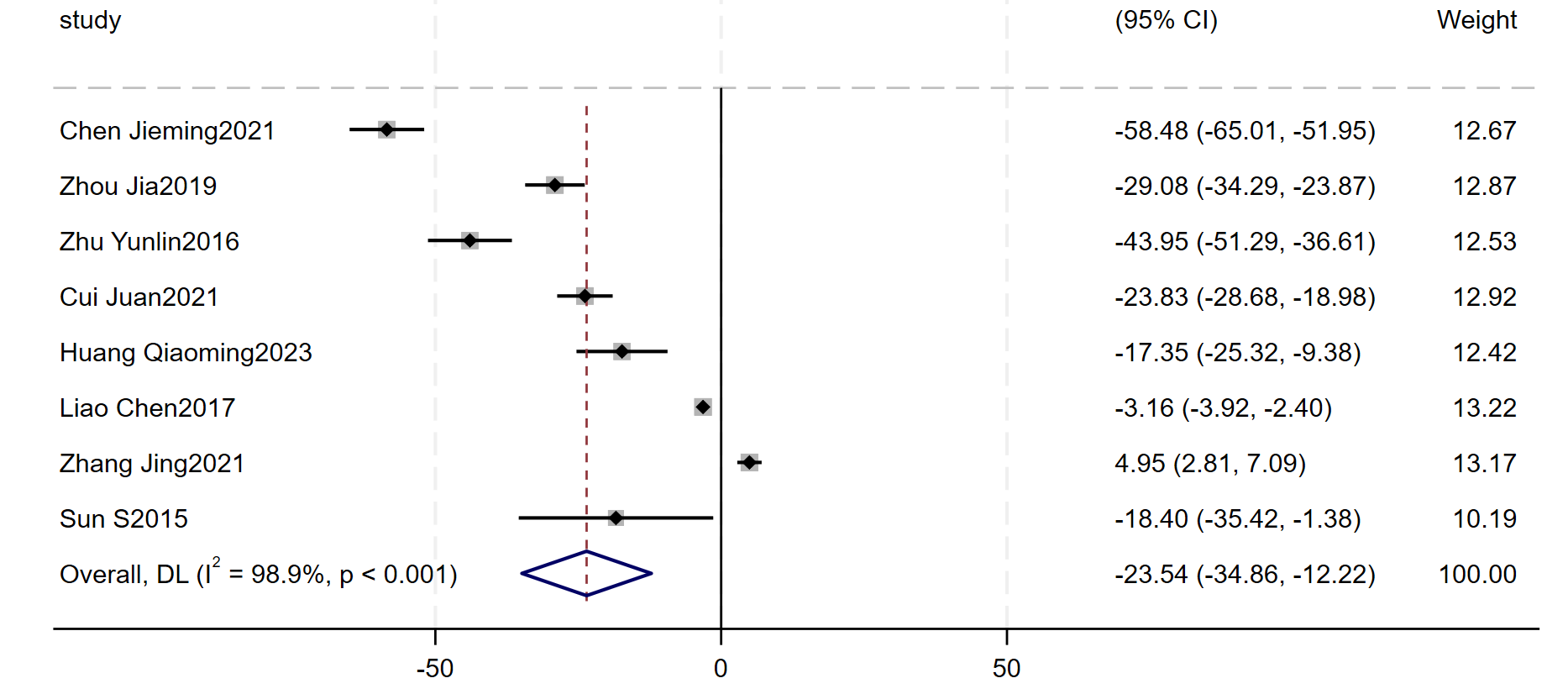


**Supplementary Figure 13.** Meta analysis of CRRT versus Non-blood purification group in IL-6.


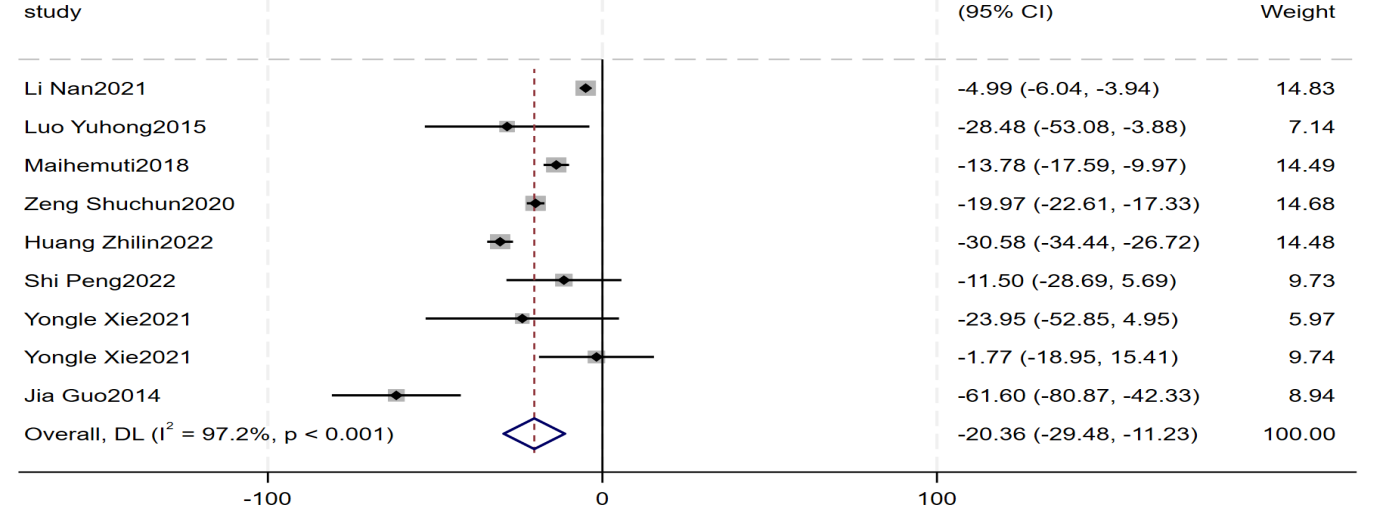


**Supplementary Figure 14.** Meta analysis of CRRT versus Non-blood purification group in IL-6 after excluding the study by Li Nan.


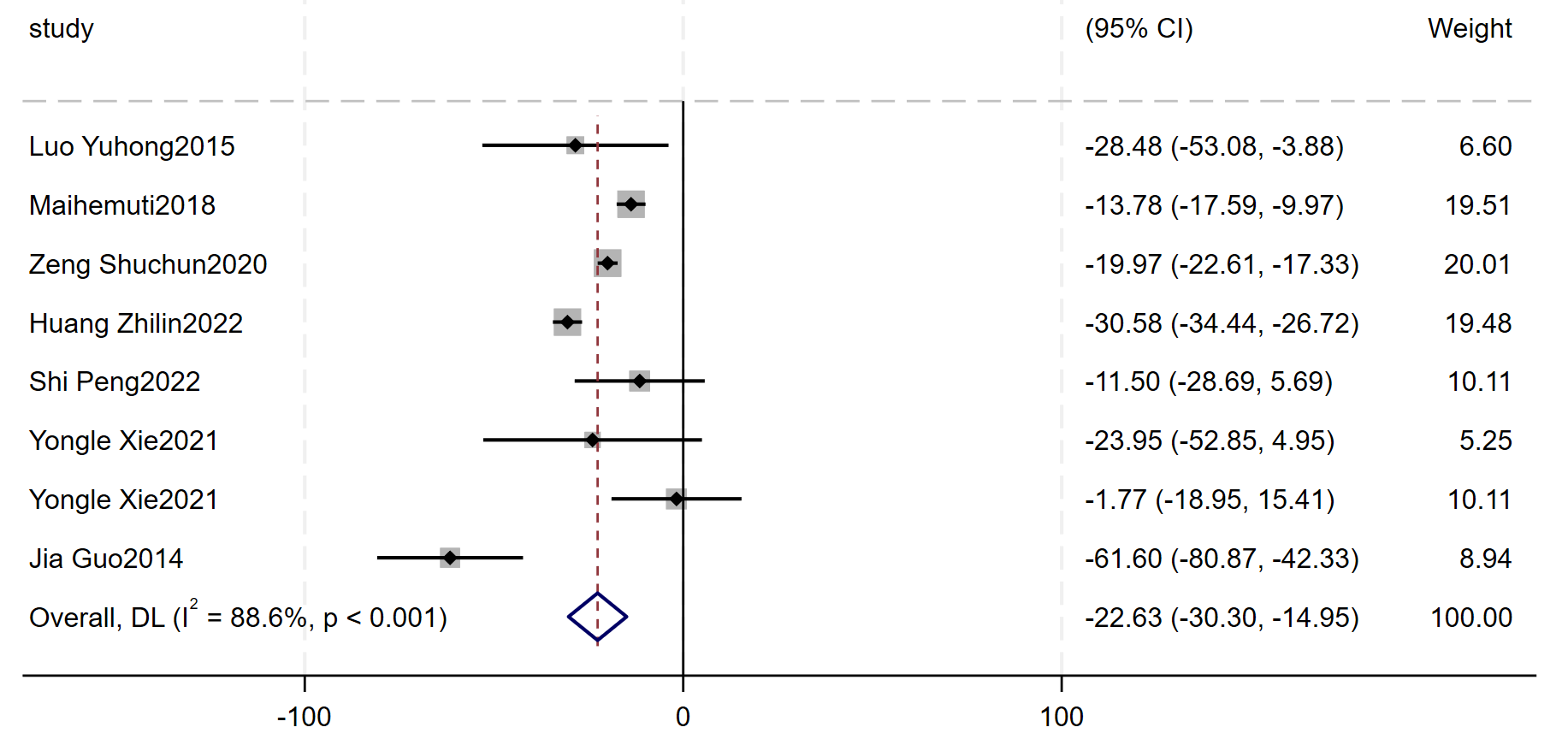


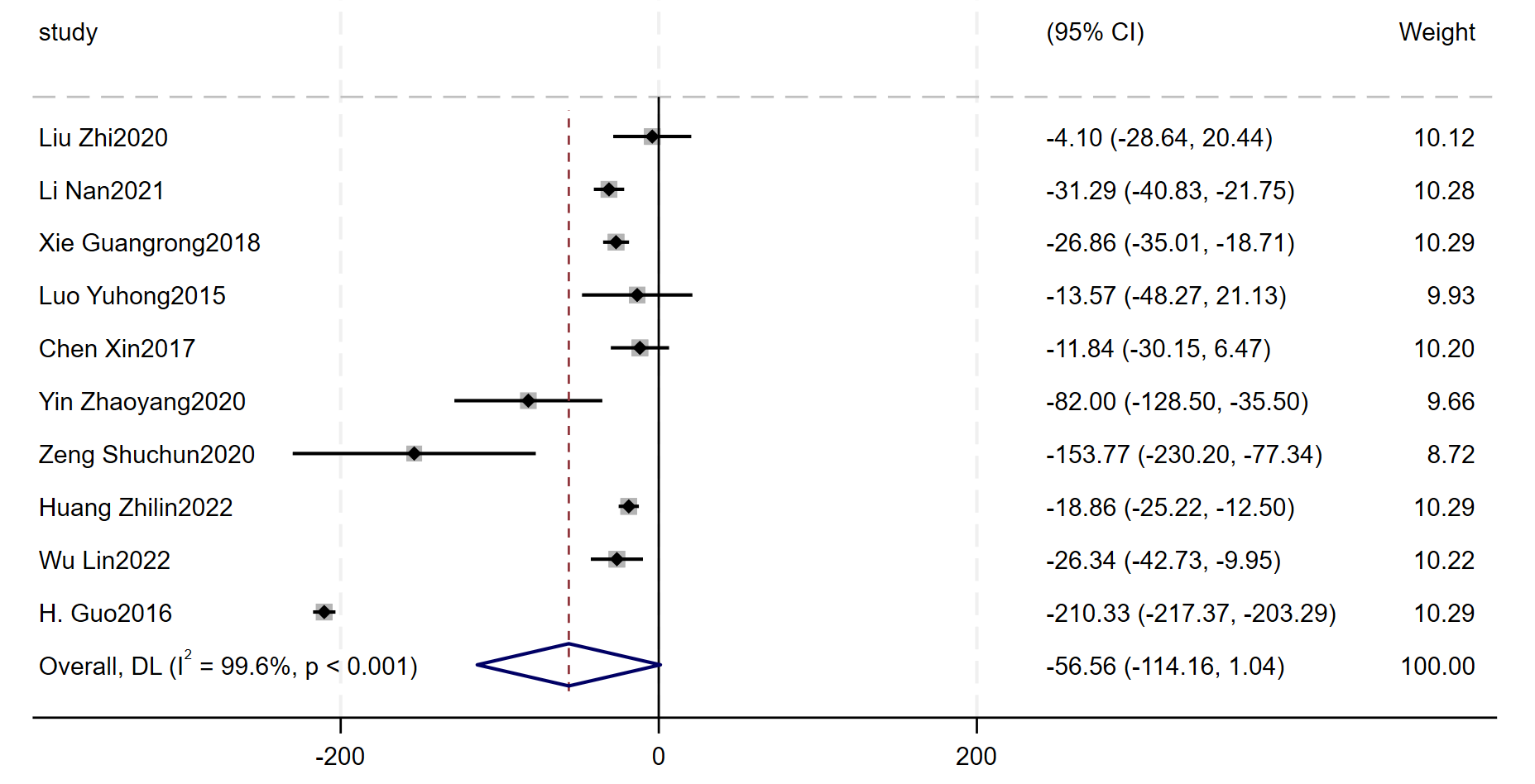
**Supplementary Figure 15.** Meta analysis of CRRT versus Non-blood purification group in Scr.

**Supplementary Figure 16.** Meta analysis of CRRT versus Non-blood purification group in Scr after excluding the study by H. Guo.


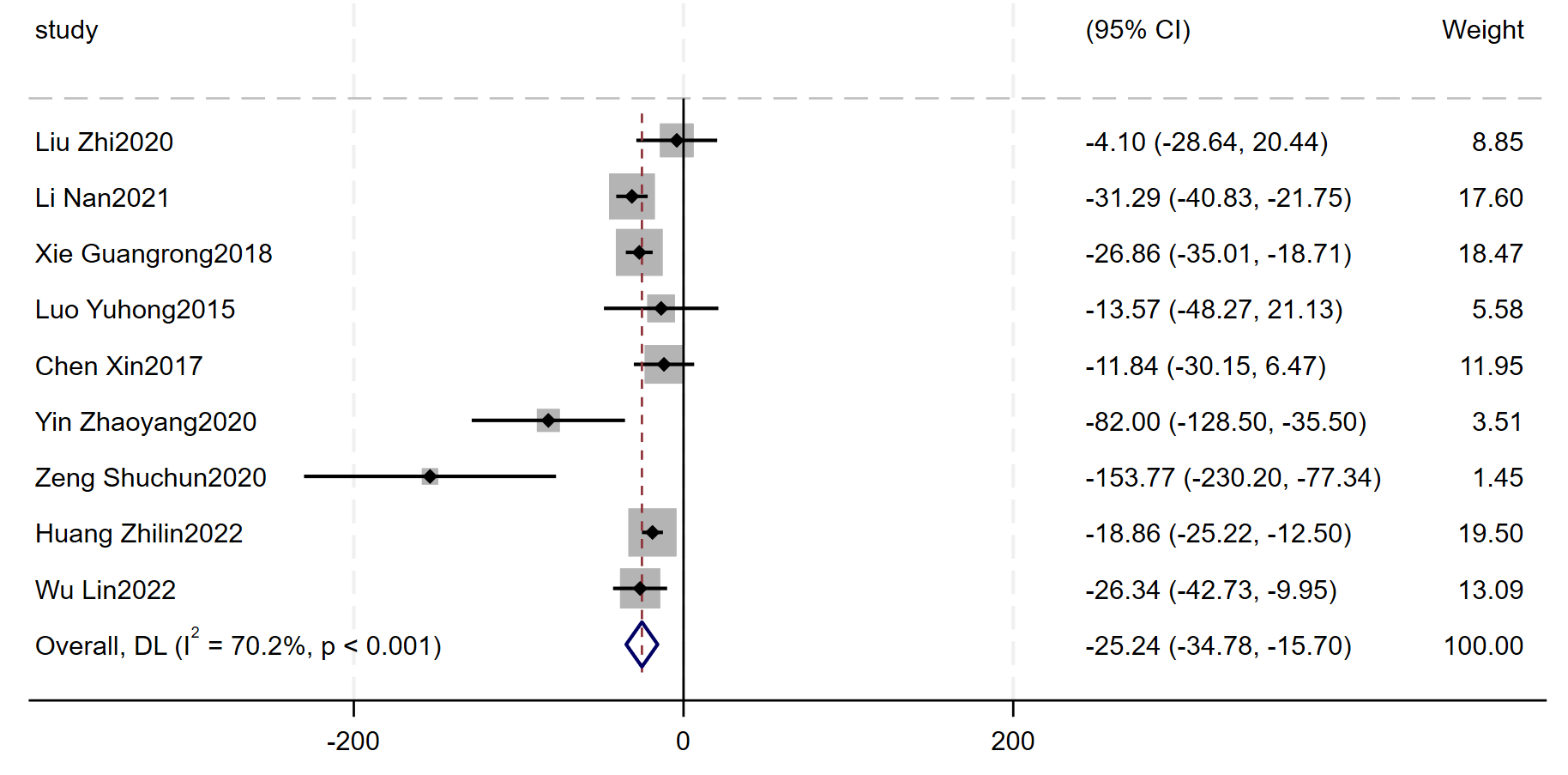


**Supplementary Figure 17.** Meta analysis of Hemoperfusion combined with CRRT versus Non-blood purification group in Scr.


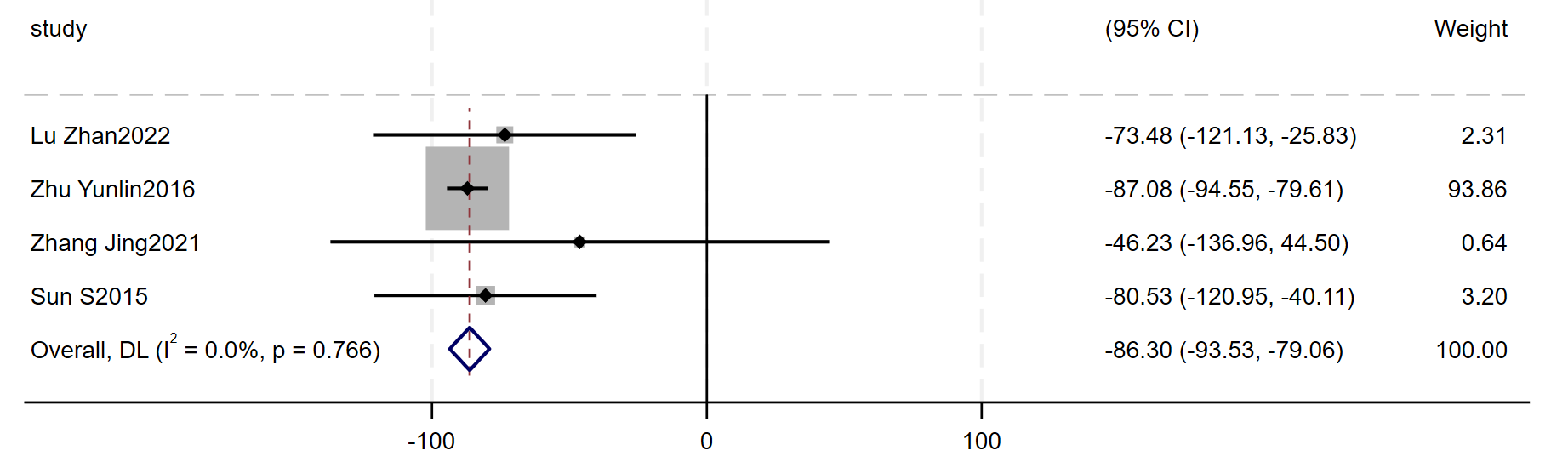


**Supplementary Figure 18.** Meta analysis of Hemoperfusion combined with CRRT versus CRRT in Scr.


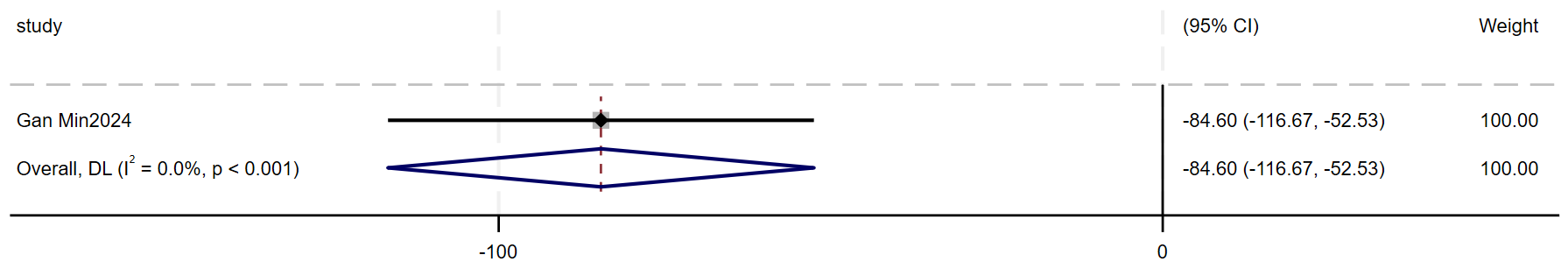


**Supplementary Figure 19.** Meta analysis of Hemoperfusion combined with CRRT versus Non-blood purification group in Mortality.


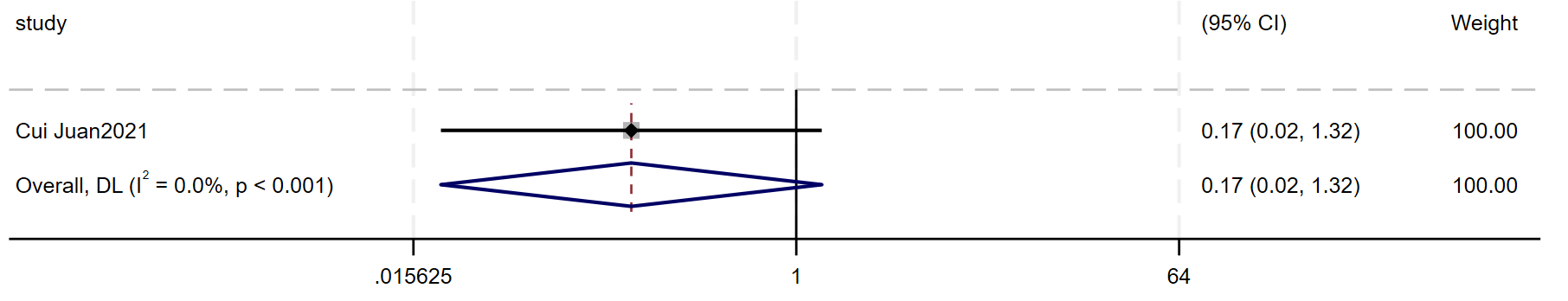


*Note: Lu Zhan 2022 was not displayed in the forest plot due to zero events in both arms.*

**Supplementary Figure 20.** Meta analysis of Hemoperfusion combined with CRRT versus CRRT in Mortality.


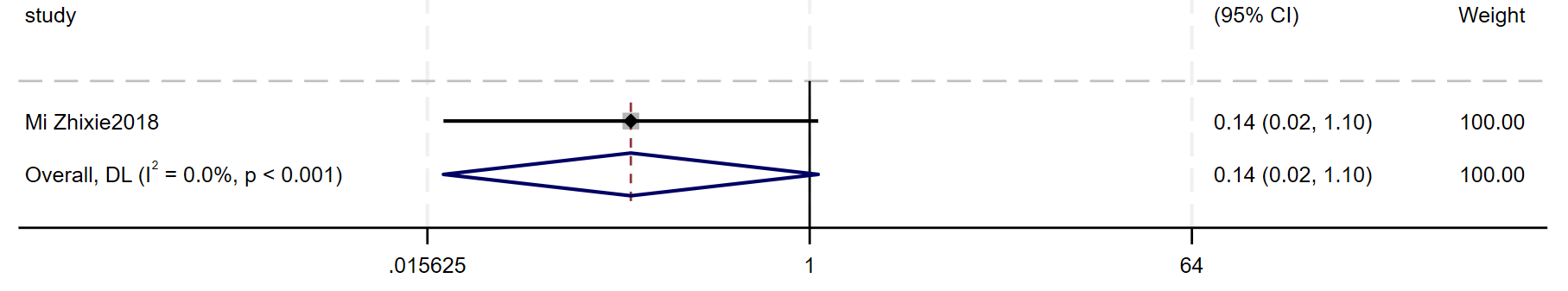


**Supplementary Figure 21.** Meta analysis of CRRT versus Non-blood purification group in Mortality.


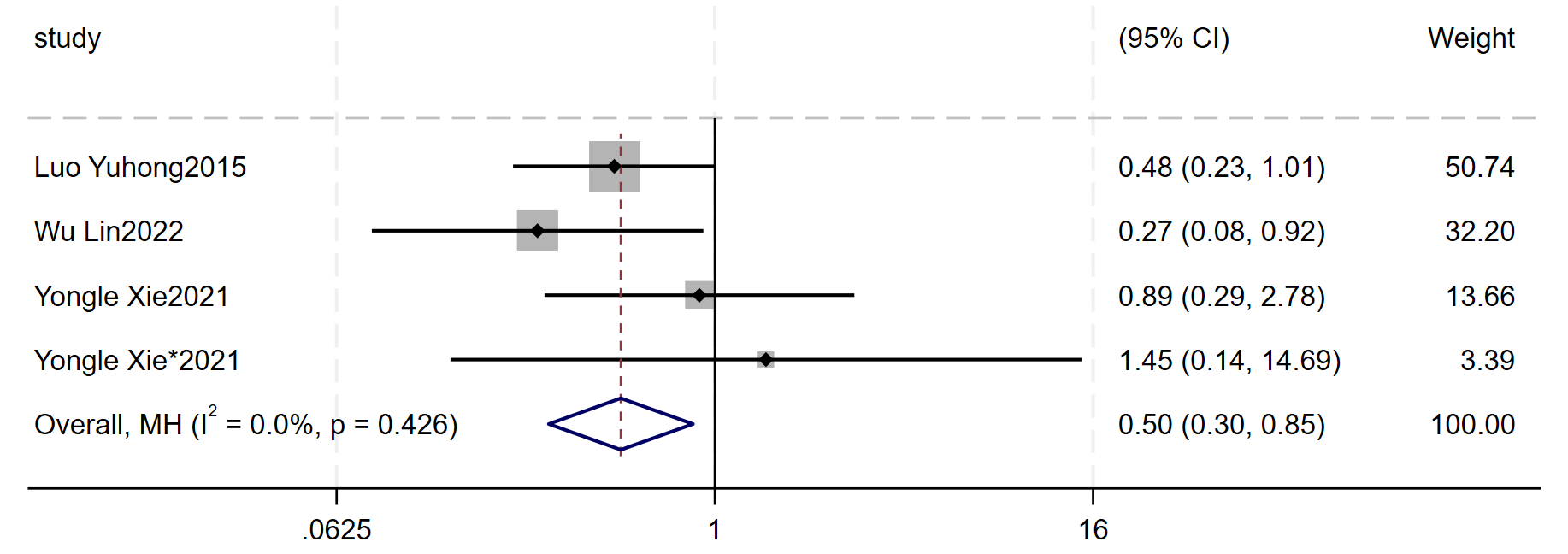


**Supplementary Table 14**. SUCRA Ranking for clinical efficacy of different blood purification modes on SAP

| **Treatment** | **AMS** | | **AMS2** | | **CRP** | | **CRP2** | | **IL-6** | | **Scr** | | **APEⅡ** | | **TG** | | **Mortality** | |
| --- | --- | --- | --- | --- | --- | --- | --- | --- | --- | --- | --- | --- | --- | --- | --- | --- | --- | --- |
|  | **SUCRA** | **Rank** | **SUCRA** | **Rank** | **SUCRA** | **Rank** | **SUCRA** | **Rank** | **SUCRA** | **Rank** | **SUCRA** | **Rank** | **SUCRA** | **Rank** | **SUCRA** | **Rank** | **SUCRA** | **Rank** |
| C | 36.1% | 4 | 36.8% | 4 | 42.8% | 4 | 46.5% | 4 | 44.7% | 5 | 54.7% | 3 | 64.4% | 2 | 47.9% | 3 | 62.8% | 3 |
| C+PE | 79.4% | 1 | 80.9% | 1 | 97.4% | 1 | 73.2% | 2 | 86.5% | 1 | / | / | / | / | 68.4% | 2 | / | / |
| HD | / | / | / | / | 20.7% | 5 | 21.8% | 5 | / | / | 39.6% | 4 | / | / | / |  | 5.8% | 6 |
| HDF | / | / | / | / | / | / | / | / | 66.9% | 4 | / | / | / | / | / | / | / | / |
| HF | / | / | / | / | / | / | / | / | 27.4% | 7 | 37.9% | 5 | 39.2% | 3 | / | / | / | / |
| HP+C | 74.3% | 2 | 78.6% | 2 | 75.1% | 2 | 88.3% | 1 | 67.9% | 3 | 61.2% | 2 | 99.0% | 1 | 83.7% | 1 | 88.8% | 1 |
| HP+HD | / | / | / | / | 55.8% | 3 | 62.5% | 3 | / | / | 84.2% | 1 | / | / | / | / | / | / |
| HP+HDF | / | / | / | / | / | / | / | / | 78.8% | 2 | / | / | / | / | / | / | / | / |
| N | 0.8% | 5 | 0.8% | 5 | 8.2% | 6 | 10.9% | 6 | 13.8% | 9 | 22.5% | 6 | 13.5% | 5 | 11.6% | 5 | 35.6% | 4 |
| PE | / | / | / | / | / | / | / | / | 38.1% | 6 | / | / | 33.9% | 4 | 38.4% | 4 | 22.9% | 5 |
| PE+HD | 59.3% | 3 | 53.0% | 3 | / | / | / | / | 25.9% | 8 | / | / | / | / | / | / | 84.0% | 2 |

*Note：SUCRA: Surface Under Cumulative Ranking Curve, range 0-100%; higher values indicate better efficacy). AMS2 and CRP2 mean SUCRA values after excluding study at high risk of bias.*

*Continuous renal replacement therapy (C), Continuous renal replacement therapy + Plasma exchange (C+PE), Hemodialysis (HD), Hemodiafiltration (HDF), Hemofiltration (HF), Hemoperfusion + Continuous renal replacement therapy (HP+C), Hemoperfusion + Hemodialysis (HP+HD), Hemoperfusion + Hemodiafiltration (HP+HDF), Non-blood purification group (N), Plasma exchange (PE), Plasma exchange + Hemodialysis (PE+HD)*
